# Epidemiology and Ecology of Toscana Virus Infection and Its Global Risk Distribution

**DOI:** 10.3390/v17010015

**Published:** 2024-12-25

**Authors:** Xue-Geng Hong, Mei-Qi Zhang, Fang Tang, Si-Hui Song, Jia-Yi Wang, Zhen-Yu Hu, Li-Ming Liu, Xiao-Ai Zhang, Yi Sun, Li-Qun Fang, Wei Liu

**Affiliations:** 1 State Key Laboratory of Pathogen and Biosecurity, Academy of Military Medical Science, Beijing 100071, China; hongxuegeng@foxmail.com (X.-G.H.); ic577703@163.com (M.-Q.Z.); ssh13172024@163.com (S.-H.S.); wang_jy0730@163.com (J.-Y.W.); babylovehopi@163.com (X.-A.Z.); sunyi7310@sina.com (Y.S.); 2 The 960th Hospital of the PLA Joint Logistics Support Force, Jinan 250031, China; 3 Institute of Medical Prevention and Control of Public Health Emergencies, Characteristic Medical Center of the Chinese People’s Armed Police Force, Beijing 102613, China; tf4065@163.com; 4 School of Public Health, The First Affiliated Hospital of Anhui Medical University, Hefei 230032, China; h19980706@163.com; 5 College of Animal Science and Technology, Jilin Agricultural Science and Technology University, Jilin 132101, China; aliuliming1984@126.com

**Keywords:** Toscana virus, sandfly, *Phlebovirus*, machine learning

## Abstract

Toscana virus (TOSV), a member of the *Phlebovirus* genus transmitted by sandflies, is acknowledged for its capacity to cause neurological infections and is widely distributed across Mediterranean countries. The potential geographic distribution and risk to the human population remained obscure due to its neglected nature. We searched PubMed and Web of Science for articles published between 1 January 1971 and 30 June 2023 to extract data on TOSV detection in vectors, vertebrates and humans, clinical information of human patients, as well as the occurrence of two identified sandfly vectors for TOSV. We further predicted the global distribution of the two sandfly vectors, based on which the global risk of TOSV was projected, after incorporating the environmental, ecoclimatic, biological, and socioeconomic factors. A total of 1342 unique studies were retrieved, among which 389 met the selection criteria and were included for data extraction. TOSV infections were documented in 10 sandfly species and 14 species of vertebrates, as well as causing a total of 7571 human infections. The occurrence probabilities of two sandfly vectors have demonstrated the greatest contributions to the potential distribution of TOSV infection risk. This study provides a comprehensive overview of global TOSV distribution and potential risk zones. Future surveillance and intervention programs should prioritize high-risk areas based on updated quantitative analyses.

## 1. Introduction

Toscana virus (TOSV) belongs to the species *Phlebovirus toscanaense* within the genus *Phlebovirus*, which is part of the family Phenuiviridae, order Hareavirales [1], that is transmitted to humans through the bite of phlebotomine sandflies. The virus was first identified in 1971 from *Phlebotomus* (*P.*) *perniciosus* in central Italy [2,3]. Almost a decade later in 1984–1985, TOSV infection was first shown to cause human disease in individuals who traveled from Portugal and Italy back to Sweden and the United States, respectively [4,5]. Human cases of TOSV infection have been reported from countries within the Mediterranean basin, with recent cases also reported in North Africa and the Balkan Peninsula [6]. In regions where it is endemic, TOSV represents a significant etiology of viral meningitis during the warm season. For example, in southeastern France, TOSV has been identified as one of the three most prominent causes of aseptic meningitis alongside enteroviruses and herpesviruses [7]. Similarly, in central Italy, TOSV has surpassed enteroviruses to become the primary cause of viral meningitis [8]. The majority of individuals infected with TOSV are asymptomatic and do not seek medical assistance [9]. Among those presenting symptoms, non-specific manifestations such as febrile illness accompanied by headache, nausea, or vomiting are frequently observed. These patients are likely to remain undiagnosed or misdiagnosed without laboratory confirmation. Given its clinical resemblance to other viral infections [6,7,10], the diagnosis of TOSV heavily relies on laboratory tests including molecular or serological antibody assays such as enzyme-linked immunosorbent assay (ELISA) and immunofluorescence assay (IFA) [7]. However, the reliability of TOSV diagnosis using serological methods is limited due to potential cross-reactivity with co-circulating phleboviruses in certain countries [11,12].

Despite the increasing recognition of TOSV’s medical significance within the European Union (EU)/European Economic Area (EEA), it is not listed as a notifiable disease for active surveillance, and no official case definition has been suggested. Several sandfly species have been implicated, or suggested as vectors of TOSV, with two species, *P. perniciosus* and *P. perfiliewi* relevant for transmission to humans [13,14,15,16,17]. No animal reservoir has yet been identified, although serological positive results have been obtained in domestic animals such as dogs, cows, and sheep [18,19,20,21]. The potential of TOSV for geographic spread, increasing the likelihood of its emergence in new areas, underscores the necessity to investigate its geographic distribution and to explore the risk of TOSV infection in high-risk areas for disease transmission. Here through an exhaustive literature search, we have compiled a comprehensive database on TOSV gathered since 1971, with a specific focus on TOSV geographic distribution in humans, vectors, and vertebrates, as well as prediction of TOSV infection while demonstrating the significant factors that may shape their distribution. This knowledge might help provide guidance to health authorities in determining surveillance priorities and prioritizing preventive measures.

## 2. Materials and Methods

### 2.1. Data Collection and Management

**Figure 1:**
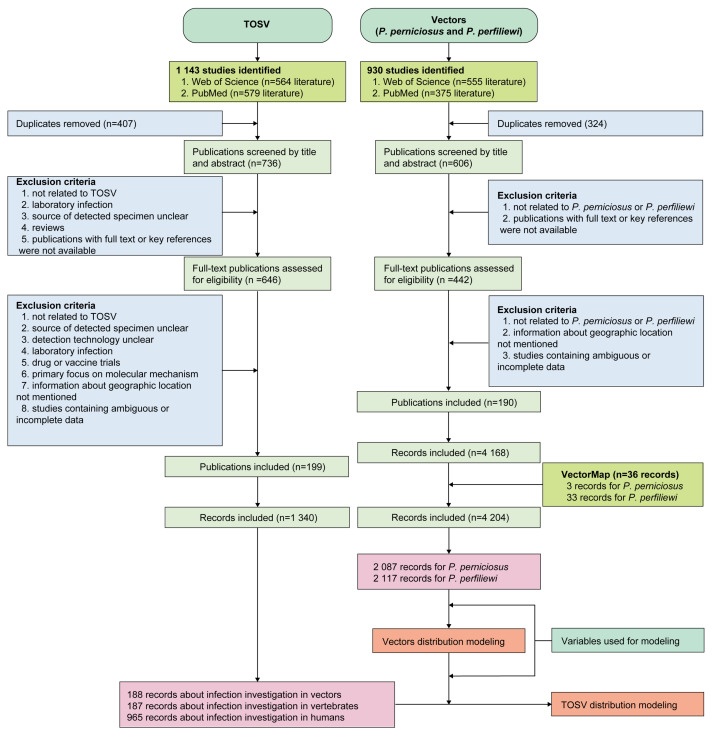
Flowchart of the literature review process.

We conducted an extensive search on PubMed and Web of Science to identify articles reporting the detection of TOSV in field investigation or clinical settings using the specific search terms “Toscana virus” OR “TOSV”. The search covered articles published from January 1, 1971 to June 30, 2023. Additionally, we used the term “*Phlebotomus perniciosus*” OR “*Phlebotomus perfiliewi*” OR “*P. perniciosus*” OR “*P. perfiliewi*” to identify sandfly-related publications from 1 January 2002 to 30 June 2023. Articles on sandfly vectors were restricted to those published after 2002 due to the abundance and quality of literature in recent decades and advancements in species identification technology that have improved precision. Language restrictions were not imposed during the search process. A two-step screening approach was employed: an initial review of the title or abstract, followed by a thorough evaluation of the full text for relevant papers. Data on four types of TOSV infection events were collected: case reports documenting acute TOSV infection in humans; seroprevalence studies conducted in human populations; seroprevalence studies carried out in non-human vertebrate populations; and detection of TOSV RNA in wild-caught sandflies or domestic and wild animals by molecular assay with or without virus isolation results. Studies that lacked clear and unambiguous descriptions of the detection methods employed were excluded. Specifically, studies that did not explicitly specify the use of molecular techniques such as PCR or serological assays like ELISA, or any other methods that were not adequately defined, were not included. Additionally, our analysis excluded experimental inoculation of TOSV in laboratory settings, drug or vaccine trials, studies on molecular mechanisms, studies without geographical information, with inaccessible full texts or key references, and studies containing ambiguous or incomplete data, such as datasets lacking critical details like sample sizes, or datasets with inconsistencies in the reported results (Figure 1). The inclusion and exclusion criteria are further detailed in Table S1. The extracted information regarding TOSV and sandfly vectors was recorded in separate databases. Additionally, sandfly distribution data were supplemented by information from VectorMap [22]. All articles underwent independent review by two authors (MQ Zhang and SH Song), with any disagreement resolved by a third author (XG Hong). For human infections, we differentiated between confirmed human infections and serological positives. Following the USA Centers for Disease Control and Prevention (CDC) 2015 case definition criteria for arbovirus infection [23], confirmed human infections were defined as meeting at least one of the following criteria: virus isolation or positive detection of specific viral antigen/nucleic acid; four-fold or greater change in virus-specific antibody titers/seroconversion between acute and convalescent sera; presence of virus-specific IgM/IgG antibodies in serum with confirmatory virus-specific neutralizing antibodies; positive detection of virus-specific IgM antibodies in cerebrospinal fluid (CSF) with or without reported pleocytosis, but negative result for CSF IgM antibodies against other region-endemic arboviruses. Serological positive refers to individuals testing positive for antibodies, excluding situations that fall within the definition of confirmed human infections. Details on the qualified detection methods used for these infection events are provided in Table S2. We also comprised a dataset on ecological factors for modeling the distribution risk of vector and TOSV infection. These variables include data on animal, environment, land use, and human activity that are known or hypothesized to contribute to sandfly vectors’ ecological suitability and affect TOSV infection risk [2,16,21,24,25,26,27,28,29,30,31]. Tables S3 and S4 provide details on the sources, spatial resolutions, and time spans of the variables. Further information on data extraction, geo-positioning of occurrence data, and assembling occurrence data and covariates are given in Text S1.

### 2.2. Analysis of the Distribution of TOSV Infections in Humans and Hosts

We extracted demographic characteristics and clinical manifestations of confirmed TOSV infections in humans. We mapped the distribution and frequency of local human infections and noted the earliest report date from each country. We also extracted information on local and imported confirmed human infections, as well as serological positives. For imported cases, we documented their routes of importation. The various species of arthropods and vertebrates that carry TOSV were identified. When mapping the distribution of infections, a distinction was made between point-based and area-based data for all TOSV infections (Text S1).

### 2.3. Ecological Modeling Analysis of Sandfly Vectors

The Boosted Regression Trees (BRT) algorithm was employed to conduct ecological modeling of the confirmed sandfly vectors of TOSV, namely, *P. perniciosus* and *P. perfiliewi*. This approach effectively investigates the association between sandfly vector occurrences and a wide range of ecological predictors, encompassing 39 variables related to animals, environment, land use, and human activity. The mean values of these variables were calculated over the study duration and utilized as predictors in our ecological modeling analysis [32]. The means of variables with raw data provided at a higher resolution than the study grid (10 km × 10 km) were calculated to match the desired resolution. The geographical range for this study was defined based on observed distribution patterns in collected data, spanning latitudes 10° N and 65° N and longitudes 20° W and 70° E. Pseudo-absence locations were sampled more than 200 km away from occurrence locations using a ratio of 3:1 [33,34]. In addition to a random selection of pseudo-absence locations, we also included all absence locations reported in published sources in our models.

We randomly split the data into training (80%) and testing sets (20%). Subsequently, we fitted a model for each target species of sandfly [32,35,36]. This process was independently repeated 100 times, resulting in an ensemble of 100 models based on resampled training datasets. Receiver operating characteristic (ROC) curves were used to calculate the average test area under the curve (AUC) for the test sets. The relative contribution (RC) of each predictor, model fit values, and AUC value were then averaged across the ensemble of 100 models to represent the final estimation and predictive performance. By utilizing the Youden index derived from the average ROC curve across all 100 models in the ensemble, we determined an optimal threshold value for predicting sandfly occurrence (detailed in Text S2).

### 2.4. Ecological Modeling Analysis of TOSV Infection

The predicted probabilities of the presence of two sandfly vectors were utilized as predictors in the ecological modeling analysis of TOSV, along with the same variables used in previous vector modeling. The same ecological model and performance evaluation methods were also applied to predict TOSV infection. Locations, where positive serological tests from humans did not meet the criteria for confirmed infection, were excluded from our modeling analysis due to the potential cross-reactivity among phleboviruses. However, if these locations coincided with the selected pseudo-absences for our study, they were excluded from our modeling validation due to their inferred higher risk of TOSV occurrence (Text S2).

The model’s outcome predicted potential risk areas for TOSV infection by integrating vectors’ influence with other predictors. By utilizing the Youden index, we determined the optimal threshold value for predicting TOSV occurrence. Furthermore, we mapped the potential distribution of TOSV and calculated and presented the affected area and population at potential risk within the study area.

## 3. Results

### 3.1. Data Retrieval and Assembly

We identified a total of 1143 studies on TOSV infection and 930 studies on sandfly vectors. Among them, 199 studies on TOSV infection (1340 records) and 190 studies on sandfly species (4168 records) met the inclusion criteria (Figure 1, Table S1). Additionally, we retrieved an additional 36 records from VectorMap on sandfly species occurrence. This process resulted in a final dataset encompassing 1340 records on TOSV and 4204 records on sandfly species.

**Figure 2:**
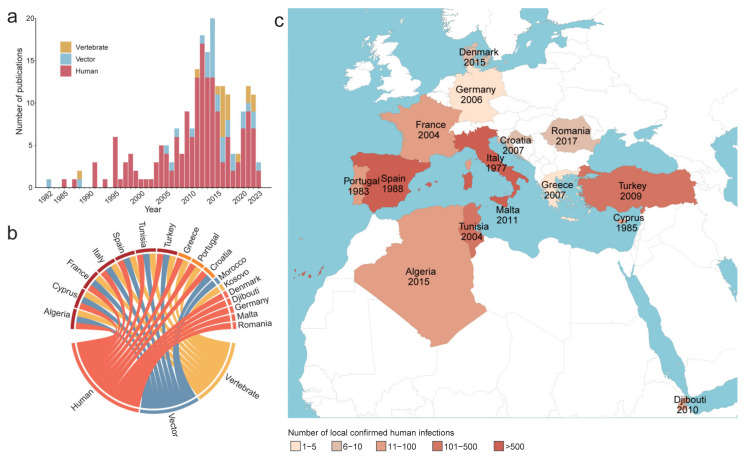
Publications on TOSV detection in humans, vectors, and vertebrates collected up to June 2023. (**a**) Annual distribution of TOSV-related publications by host type. (**b**) Reported countries with confirmed TOSV infections in vertebrates, vectors, and human beings. (**c**) Distribution of local confirmed human infections. The year indicated beneath each country’s name represents the earliest documented instance of confirmed infections in humans, as derived from published sources. For human patients, only those with laboratory-confirmed infections were included. TOSV, Toscana virus.

The first identification of TOSV infection in *P. perniciosus* was documented in 1971; however, there were only sporadic reports until 1995. Subsequently, the number of publications on TOSV infection has steadily increased, reaching a total of 199 publications (Figure 2a). Most of these publications focused primarily on the detection of TOSV among humans (79.9%), while 16.1% reported its detection among vectors and 9.0% involved vertebrates. Nine publications reported TOSV infection in multiple host types. As of 30 June 2023, a laboratory-confirmed TOSV infection has been reported in 17 countries. Among these countries, Algeria, Cyprus, France, Italy, Spain, Tunisia, and Turkey have reported TOSV infection in both human populations, vectors, and vertebrates. Croatia has reported infection in both vectors and human populations; Greece and Portugal have reported infection in both vertebrates and human populations; while five countries (Denmark, Djibouti, Germany, Malta, and Romania) have exclusively reported human infection. Morocco and Kosovo have not yet confirmed any human infections but have exclusively reported vertebrate or vector infections (Figure 2b). Over half of the retrieved publications on TOSV infection (59.3%) documented confirmed human infections, with a higher occurrence observed in countries surrounding the Mediterranean Sea. The earliest recorded human infection can be traced back to a vector-borne virus research study conducted from 1977 to 1988 across multiple hospitals in the central region of Italy (Figure 2c) [37].

The sandfly vectors dataset included a total of 4204 records, with 2087 involving *P. perniciosus* and 2117 involving *P. perfiliew* (Figure 1). *P. perniciosus* was predominantly documented in countries located in southwestern Europe and northeastern Africa. In contrast, *P. perfiliewi* exhibited a wider distribution range, covering regions in southern Europe, northern Africa, and western Asia (Figure S1).

### 3.2. Epidemiological Features of TOSV Infections

**Figure 3:**
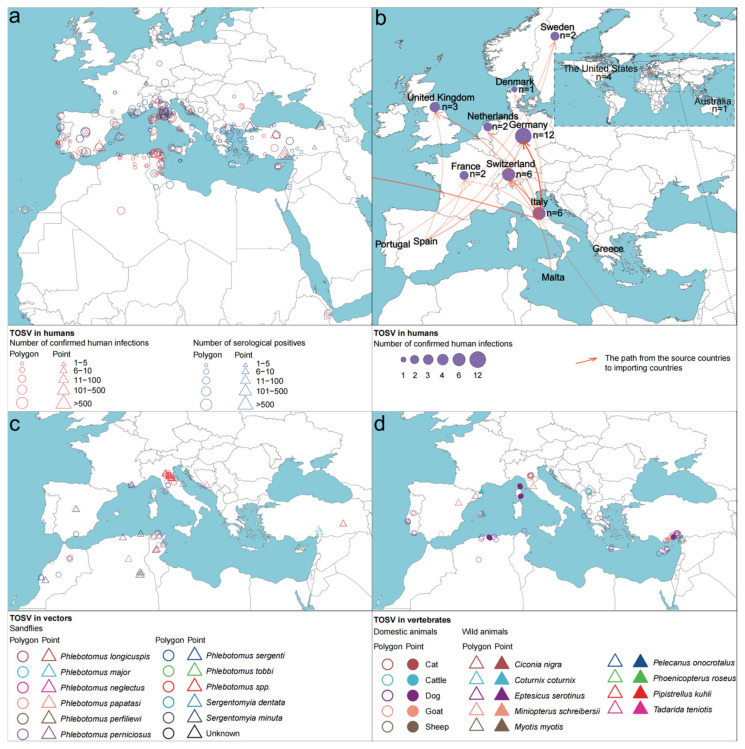
Global distribution of TOSV infections: (**a**) in humans and (**b**) human-imported cases. The purple circles represent imported cases, with the size of each circle indicating the number of imported cases, as indicated on the right side. The orange-red arrows depict the path from the source countries to importing countries, with their opacity representing the number of imported cases. (**c**) In vectors and (**d**) in vertebrates. TOSV, Toscana virus.

In total, there were 7571 reported cases of TOSV infection, including 2567 confirmed human infections and 5004 identified infections through serological tests. Confirmed human infections have been documented in 15 countries (Table 1). The majority of cases were concentrated in European and African countries surrounding the Mediterranean region. Italy had the highest number of cases (956), followed by Spain (693) and Tunisia (466). This geographic distribution closely mirrored that of individuals who tested positive through serological testing, although the latter exhibited a more extensive dispersion (Figure 3a).

Sex information was available for 732 confirmed human infections with males accounting for 55.7% and females accounting for 44.3% (Table 1). Age information was available for 515 confirmed human infections, with a mean age of 54.6 years and 30.5% being aged ≥60 years. Among the recorded occupations in the TOSV infection group, outdoor occupation type accounted for 20 cases. Notably, several cases with indoor occupations also had a history of travel or outdoor activities in endemic areas [10,38,39]. The peak occurrence of TOSV infections was observed between 2010 and 2019, with a total of reported cases reaching 1132 individuals. Italy during the 1990s and Spain during the 1980s reported the highest number of cases. Tunisia and Turkey experienced a significant number of cases between 2010 and 2019. Over time, TOSV infection has shown an increasing trend affecting more countries, especially within the Mediterranean region (Table 1).

A total of 39 imported cases of TOSV infection have been reported in 10 countries to date, with Italy being the primary source responsible for exporting 25 cases to eight countries: Germany (10), Switzerland (4), the United States (4), the United Kingdom (2), the Netherlands (2), France (1), Denmark (1), and Australia (1). Furthermore, within Italy, five cases of domestic transregional transmission have been recorded. Germany has reported 12 imported cases from two countries: Italy (10) and Spain (2). Both Italy and Switzerland had reported 6 imported cases (Figure 3b).

### 3.3. Clinical Features of TOSV Infections

The symptom profiles were obtained from 288 confirmed human infections, revealing that headache (92.7%) and fever (91.3%) were the most frequently reported symptoms. Equally prevalent were neurological manifestations (91.3%), including altered mental status, ataxia, vertigo, hearing disorders, language disorders, lethargy, neck rigidity, nystagmus, paresis, photophobia or phonophobia, hydrocephalus, seizure, meningitis, encephalitis, and meningoencephalitis. Other commonly observed non-specific symptoms included nausea or vomiting (74.0%) and ocular manifestations (34.0%). Less frequently reported symptoms included musculoskeletal, cutaneous, gastrointestinal manifestations, and systematic manifestations (Table 1). Six fatal cases have been documented, five of which occurred in a hospital located in Bucharest, Romania. The median age of the fatal patients was 78.6 years old (range: 68–91), and among them, five individuals had underlying conditions such as hypertension, diabetes mellitus, ischemic heart disease, stroke sequelae, and congestive heart failure. The remaining patient without any underlying condition was a 73-year-old German male who had recently returned from a trip to Tuscany, Italy [40,41].

### 3.4. Vectors of TOSV Infections

In total, 10 sandfly species were identified to carry TOSV, resulting in 73 positive records across nine countries. Among these species, eight belonged to the *Phlebotomus* genus; specifically, *P. perniciosus* and *P. perfiliewi* were more extensively reported and widely distributed compared to the other six species, including *P. longicuspis*, *P. major*, *P. neglectus*, *P. papatasi*, *P. sergenti*, and *P. tobbi* (Figure 3c). The remaining two species belonging to the *Sergentomyia* genus, *S. dentata* and *S. minuta*, were also found infected with TOSV in the Mediterranean Sea surrounding countries, where most human infections occurred (Figure 3a,c).

Five domestic animal species were found to harbor TOSV, with dogs accounting for 60% of publications reporting positive detections. TOSV was found in nine wild animal species, predominantly avian and chiropteran species such as birds and bats (Figure 3d). While positive detections do not necessarily imply that these vertebrates serve as reservoirs, the geographical distribution of vertebrates and vector hosts showed a high level of consistency with that of human beings, predominantly concentrated around the Mediterranean region (Figure 3a,d).

### 3.5. Ecological Modeling of Sandfly Vectors

The ensemble models demonstrated exceptional predictive performance, with AUC values derived from 100 BRT models. For sandfly vectors, the models accurately predicted *P. perniciosus* (average AUC of 0.963) and *P. perfiliewi* (average AUC of 0.978) (Table 2, Figure S2a,b). Key drivers varied between species; however, common predictors included distance from the coastline, annual temperature range, human development index, nighttime lights index, and population density—all significantly contributing to both sandfly models (RC > 5%). Notably, population density (RC of 25.3%) and human development index (RC of 14.7%) exclusively emerged as top predictors for *P. perniciosus* (Table 2, Figure S3b,c), whereas distance from the coastline (RC of 23.3%), mean temperature of the driest quarter (RC of 15.3%), and cropland coverage (RC of 14.5%) were exclusively identified as top predictors for *P. perfiliewi* (Table 2, Figure S4b,c).

### 3.6. Ecological Modeling and Risk Assessment of TOSV Infection

**Figure 4:**
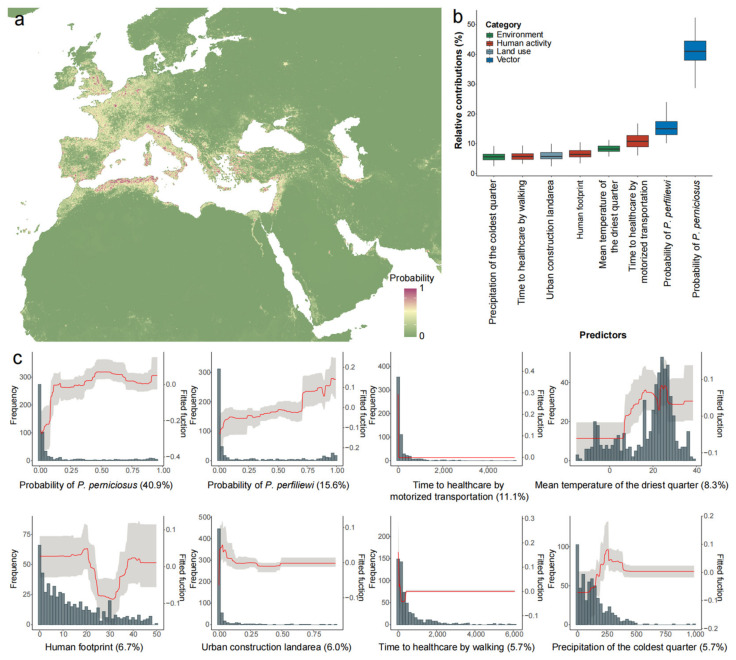
Occurrence probability of TOSV infection and relative contributions (RCs) of the significant predictors (RC > 5%) based on the BRT models: (**a**) predicted risk distribution of TOSV infection (latitude 10° N–65° N, longitude 20° W–70° E); (**b**) RCs of predictors for TOSV infection; (**c**) response curve of predictors in BRT models for TOSV infection. Mean curves (red) and 95% percentiles (grey) show the contribution to predicting the probability of occurrence. The histograms show the frequency distributions of the predictors. TOSV, Toscana virus; RC, relative contribution; BRT, Boosted Regression Tree.

By incorporating the predictive probability of sandfly vector occurrences, our modeling of TOSV infection achieved decent predictive performance, with an average AUC estimated at 0.907 (Table 2, Figure S2c). We estimated both the population size and geographical range that could potentially be affected by TOSV and its related vectors, surpassing the extent reported in previous publications (Figure 4a, Figures S3a and S4a). Europe was projected to have the broadest geographic range (887.7 thousand km^2^, 596.1–1215.7) and the largest population size (332.3 million people, 261.5–392.3) potentially affected by TOSV, particularly in Southern and Western Europe. Africa was also projected with an expanded geographical range, covering 233.2 thousand km^2^ (170.0–316.2), also with an enlarged population size of 158.5 million (124.5–210.0) at risk of TOSV. Within Africa, the largest area and population at risk were observed in Northern Africa, spanning an area of 223.8 thousand km^2^ (167.6–293.6), comprising 136.6 million people (109.3–174.3) (Table 3). Moreover, Asia is projected to cover a similarly extensive region around 223.5 thousand km^2^ (164.9–282.8), with a population size of approximately 208.7 million people (181.1–235.7), while Western Asia has taken the most part of the area (193.9 thousand km^2^, 148.3–236.7) and population at risk (154.8 million, 138.7–168.9 people) (Table 3). The two sandfly vectors had the most significant impact as predictors, with RC estimates of 40.9% and 15.6%, respectively. Time to healthcare by motorized transportation (RC of 11.1%) and mean temperature of the driest quarter (RC of 8.3%) were followed in importance (Table 2, Figure 4b,c).

## 4. Discussion

Although TOSV is an important cause of meningitis and encephalitis, it remains a neglected virus with limited published data. Here we projected that the TOSV endemic area is much larger than believed, which was significantly associated with the occurrence probabilities of *P. perniciosus* and *P. perfiliewi*. Both sandfly species also present a significantly larger potential risk area in comparison to the field survey data. Considering the substantial involvement of sandfly vectors in spreading TOSV infection, along with the absence of specific vaccines or treatments for this infection, vector control emerges as the most practical approach to managing TOSV infections. Besides vector factors, human activities significantly contribute to the distribution patterns of TOSV infection. For instance, there exists a substantial negative correlation between time to healthcare by motorized transportation (RC of 11.1%) and the predicted risk values of TOSV infection (Figure 4c), implying the likelihood of underdiagnoses or misdiagnoses for TOSV infection, particularly in remote rural areas where laboratory testing capacities are insufficient.

We projected that the areas of TOSV infection risk are primarily located in Southern Europe, Western Europe, North Africa, and Western Asia. Within Africa, Northern Africa represents the largest area at risk, covering a span of 223.8 thousand km^2^ (167.6–293.6) (Table 3). Studies performed in Northern Africa (Algeria and Tunisia) showed that the percentage of the population that possess neutralizing antibodies against TOSV (22–33%) is much higher than that observed in southwestern Europe [6]. These data align with our current prediction that TOSV infection has been remarkably underestimated in Northern Africa.

Until recently, there was a lack of a comprehensive study with a large sample size to explore the clinical presentation of TOSV infection. Through an integrative collection of clinical data, we have now provided extensive knowledge on the symptomatic patients. It has been determined that fever, headache, and neurological symptoms involving both the central and peripheral nervous systems are commonly observed in affected individuals. TOSV should be considered as an etiological agent of aseptic meningitis with priority and include laboratory screening in cases with compatible epidemiological data.

The peak occurrence of confirmed TOSV infections was observed between 2010 and 2019 (Table 1), which may be associated with climate changes, improvements in healthcare systems, and advancements in surveillance and laboratory testing. However, only two cases of human TOSV infections were reported between 2020 and 2023. Despite the shorter data collection period, this figure is markedly lower compared to the corresponding period from 2010 to 2019. It is possible that diminished surveillance efforts and reallocation of healthcare resources, along with reduced human mobility due to the COVID-19 pandemic, have influenced both the reporting of TOSV infections and its transmission dynamics in subsequent years, potentially advancing the observed peak to an earlier timeframe. Notably, with increased attention to TOSV surveillance, the end of the COVID-19 pandemic, and further improvements in detection technologies, a future resurgence in TOSV infections remains a possibility.

In recent decades, globalization has increased international population movement, leading to a significant change in the global disease landscape. The dissemination of invasive species and pathogens through travel and trade as a consequence of globalization has had a profound impact on public health worldwide. A paradigmatic example is the expansion of West Nile virus into new endemic regions due to the invasion of competent vectors and the introduction of virus into areas with abundant hosts and vectors [42]. Both scenarios have been observed in the case of TOSV infection, where sandfly vectors are projected to expand their geographical range due to climate change or land use modification. Early human infections are primarily imported from endemic regions, potentially attributed to international trade or travel. However, a substantial increase in local TOSV cases has been documented in recent years. Two primary sandfly species responsible for TOSV transmission were predicted to expand beyond their original endemic areas towards suitable habitats, where local TOSV infections were elicited. Updated knowledge of the geographic distribution of TOSV needs to be further explored by combining data from human and non-human vertebrate populations based on entomological, viral, and seroprevalence aspects.

Our study has certain limitations. The variability in field detection and reporting of TOSV infection and its vectors varies across different countries and regions. This variation may introduce bias into our analysis due to limited laboratory testing and reporting capabilities in many low-income countries. Consequently, some locations classified as pseudo-absent might actually be false negatives, suggesting a potential underestimation of risk. Moreover, considering that our data span over 50 years, it is subject to flaws resulting from changing standards used for curated data. This introduces bias when applying predictive models.

## 5. Conclusions

Our study provides a comprehensive dataset on the global occurrence of TOSV infection and its vectors. We have generated risk maps for the distribution of TOSV infection, revealing various factors that could drive TOSV infection and highlighting the pivotal role played by sandfly vectors in shaping the viral infection risk landscape. It is essential to maintain continuous surveillance of field vectors/vertebrates and enhance diagnostic efforts for human patients. Measures to control sandfly vectors should be stressed with particular attention given to regions at risk of TOSV according to the current prediction.

## Figures and Tables

**Table 1:** Demographic characteristics and clinical manifestations of confirmed TOSV infections in humans.

Characteristics	All	Italy	Spain	Tunisia	Turkey	Cyprus	France	Algeria	Portugal	Djibouti	Others ^&^
Overall *	2567	956	693	466	138	95	74	49	39	30	27
Sex ^#^	733	507	43	–	37	–	74	23	30	–	19
Male	408 (55.7%)	295 (58.2%)	24 (55.8%)		18 (48.6%)		33 (44.6%)	14 (60.9%)	12 (40.0%)		12 (63.2%)
Female	325 (44.3%)	212 (41.8%)	19 (44.2%)		19 (51.4%)		41 (55.4%)	9 (39.1%)	18 (60.0%)		7 (36.8%)
Age ^#^	515	171	195	9	37	–	43	23	25	–	12
≤17	53 (10.3%)	31 (18.1%)	5 (2.6%)	1 (11.1%)	1 (2.7%)		4 (9.3%)	7 (30.4%)	4 (16.0%)		0 (0.0%)
18–34	173 (33.6%)	19 (11.1%)	93 (47.7%)	1 (11.1%)	21 (56.8%)		19 (44.2%)	13 (56.5%)	2 (8.0%)		5 (41.7%)
35–59	132 (25.6%)	36 (21.1%)	57 (29.2%)	7 (77.8%)	10 (27.0%)		10 (23.3%)	1 (4.3%)	5 (20.0%)		6 (50.0%)
≥60	157 (30.5%)	85 (49.7%)	40 (20.5%)	0 (0.0%)	5 (13.5%)		10 (23.3%)	2 (8.7%)	14 (56.0%)		1 (8.3%)
Occupation type ^#^	33	18	1	–	3	–	10	–	–	–	1
Indoor	9 (27.3%)	7 (38.9%)	0 (0.0%)		2 (66.7%)		0 (0.0%)				0 (0.0%)
Outdoor	20 (60.6%)	7 (38.9%)	1 (100.0%)		1 (33.3%)		10 (100.0%)				1 (100.0%)
Others	4 (12.1%)	4 (22.2%)	0 (0.0%)		0 (0.0%)		0 (0.0%)				0 (0.0%)
Year of occurrence ^#^	2567	956	693	466	138	95	74	49	39	30	27
1970–1979	49 (1.9%)	49 (5.1%)	0 (0.0%)	0 (0.0%)	0 (0.0%)	0 (0.0%)	0 (0.0%)	0 (0.0%)	0 (0.0%)	0 (0.0%)	0 (0.0%)
1980–1989	566 (22.0%)	12 (1.3%)	458 (66.1%)	0 (0.0%)	0 (0.0%)	95 (100.0%)	0 (0.0%)	0 (0.0%)	1 (2.6%)	0 (0.0%)	0 (0.0%)
1990–1999	587 (22.9%)	576 (60.3%)	11 (1.6%)	0 (0.0%)	0 (0.0%)	0 (0.0%)	0 (0.0%)	0 (0.0%)	0 (0.0%)	0 (0.0%)	0 (0.0%)
2000–2009	231 (9.0%)	87 (9.1%)	46 (6.6%)	18 (3.9%)	47 (34.1%)	0 (0.0%)	13 (17.6%)	0 (0.0%)	15 (38.5%)	0 (0.0%)	5 (18.5%)
2010–2019	1132 (44.1%)	232 (24.3%)	177 (25.5%)	447 (95.9%)	91 (65.9%)	0 (0.0%)	61 (82.4%)	49 (100.0%)	23 (59.0%)	30 (100.0%)	22 (81.5%)
2020–2023	2 (0.1%)	0 (0.0%)	1 (0.1%)	1 (0.2%)	0 (0.0%)	0 (0.0%)	0 (0.0%)	0 (0.0%)	0 (0.0%)	0 (0.0%)	0 (0.0%)
Manifestations ^#^	288	182	42	1	30	–	14	–	8	–	11
Headache	267 (92.7%)	177 (97.3%)	38 (90.5%)	1	26 (86.7%)		8 (57.1%)		8		9 (81.8%)
Fever	263 (91.3%)	171 (94.0%)	38 (90.5%)	1	24 (80.0%)		11 (78.6%)		8		10 (90.9%)
Neurological manifestations ^a^	263 (91.3%)	176 (96.7%)	35 (83.3%)	1	25 (83.3%)		12 (85.7%)		5		9 (81.8%)
Nausea/vomiting	213 (74.0%)	155 (85.2%)	36 (85.7%)	1	5 (16.7%)		4 (28.6%)		7		8 (72.7%)
Ocular manifestations ^b^	98 (34.0%)	86 (47.3%)	9 (21.4%)	0	3 (10.0%)		0 (0.0%)		0		5 (45.5%)
Systemic manifestations ^c^	26 (9.0%)	12 (6.6%)	2 (4.8%)	0	6 (20.0%)		4 (28.6%)		0		0 (0.0%)
Musculoskeletal manifestations ^d^	18 (6.2%)	6 (3.3%)	0 (0.0%)	0	6 (20.0%)		3 (21.4%)		1		3 (27.3%)
Cutaneous manifestations ^e^	5 (1.7%)	2 (1.1%)	0 (0.0%)	0	0 (0.0%)		0 (0.0%)		0		2 (18.2%)
Gastrointestinal manifestations ^f^	3 (1.0%)	2 (1.1%)	0 (0.0%)	0	1 (3.3%)		0 (0.0%)		0		0 (0.0%)
Other symptoms ^g^	9 (3.1%)	5 (2.7%)	1 (2.4%)	0	1 (3.3%)		2 (14.3%)		0		3 (27.3%)

* Confirmed human infections as those meeting at least one of the following criteria: isolation of virus from, or demonstration of specific viral antigen or nucleic acid; four-fold or greater change in virus-specific quantitative antibody titers in paired sera; virus-specific IgM/IgG antibodies in serum with confirmatory virus-specific neutralizing antibodies; virus-specific IgM antibodies in cerebrospinal fluid (CSF), with or without a reported pleocytosis, and a negative result for other IgM antibodies in CSF for arboviruses endemic to the region where exposure occurred. ^#^ Infections with incomplete characteristic information were excluded, e.g., only fever or headache was mentioned without any reference to the presence or absence of other symptoms. ^&^ “Others” represents countries with fewer than 10 confirmed human TOSV infections, including Romania (n = 8), Croatia (6), Denmark (6), Greece (4), Malta (2), and Germany (1). For these countries, the proportion of symptoms was combined for analysis. ^a^ Neurological manifestations include altered mental status, ataxia, vertigo, hearing disorders, language disorders, lethargy, neck rigidity, nystagmus, paresis, photophobia or phonophobia, seizure, hydrocephalus, meningitis, encephalitis, and meningoencephalitis. ^b^ Ocular manifestations include retroorbital pain or pressure, ocular pain, and blurred vision. ^c^ Systemic manifestations include malaise, fatigue, chills, and night sweats. ^d^ Musculoskeletal manifestations include fasciitis, muscle/joint pain, or stiffness. ^e^ Cutaneous manifestations include rash. ^f^ Gastrointestinal manifestations include abdominal pain or diarrhea. ^g^ Other symptoms include anorexia, weight loss, cervical lymphadenopathy, edema, hepatomegaly, pharyngodynia, rhinorrhagia, and testicular manifestations. TOSV, Toscana virus; –, No data.

**Table 2:** BRT-estimated mean relative contributions (RCs) and AUC for TOSV and vectors.

Variable Groups	Variables	RC for *P. perniciosus* (%) ^#^	RC for *P. perfiliewi* (%) ^#^	RC for TOSV (%) ^#^
Environment	Distance from the coastline	11.4 (11.0, 11.7)	23.3 (22.7, 23.8)	–
	Annual range of temperature	8.0 (7.7, 8.3)	6.6 (6.4, 6.7)	–
	Mean temperature of the driest quarter	–	15.3 (15.1, 15.6)	8.3 (8.0, 8.5)
	Precipitation of the coldest quarter	–	5.2 (5.0, 5.4)	5.7 (5.4, 6.0)
	Precipitation of the driest month	–	6.1 (5.9, 6.3)	–
Land use	Cropland	–	14.5 (14.1, 14.8)	–
	Urbanized area	–	–	6.0 (5.7, 6.3)
Human activity	Human Development Index	14.7 (14.3, 15.0)	9.8 (9.5, 10.0)	–
	Human footprint	7.1 (6.7, 7.6)	–	6.7 (6.4, 7.0)
	Nighttime lights index	6.7 (6.4, 7.1)	8.5 (8.1, 9.0)	–
	Population density	25.3 (24.7, 25.9)	6.1 (5.6, 6.5)	–
	Time to healthcare by motorized transportation	–	–	11.1 (10.5, 11.8)
	Time to healthcare by walking	–	–	5.7 (5.5, 6.0)
Animal	Mammalian richness	7.4 (7.1, 7.7)	–	–
	Pig	11.8 (11.5, 12.1)	–	–
	Sheep	7.5 (7.3, 7.7)	4.6 (4.5, 4.8)	–
Vector	Probability of *P. perfiliewi*	–	–	15.6 (14.8, 16.3)
	Probability of *P. perniciosus*	–	–	40.9 (40.0, 41.9)
AUC		0.963 (0.938, 0.978)	0.978 (0.957, 0.989)	0.907 (0.833, 0.955)

^#^ The means (2.5–97.5% percentiles) were calculated for RCs. The symbol “–” indicates that the variable was not included in the whole model. RC, relative contribution; AUC, area under the curve; TOSV, Toscana virus; BRT, Boosted Regression Tree.

**Table 3:** Model-predicted areas and population sizes at potential risk of exposure to TOSV.

Region *	Subregion *	Area (1000 km^2^) ^#^	Population Size (Million) ^#^
Total		1344.4 (931.0–1814.7)	699.6 (567.2–838.1)
Europe		887.7 (596.1–1215.7)	332.3 (261.5–392.3)
	Southern Europe	437.0 (358.6–523.1)	129.5 (120.1–139.2)
	Western Europe	308.9 (162.8–472.2)	99.8 (66.9–128.1)
	Northern Europe	94.4 (47.1–142.1)	52.6 (41.1–61.5)
	Eastern Europe	47.4 (27.5–78.3)	50.5 (33.4–63.5)
Africa		233.2 (170.0–316.2)	158.5 (124.5–210.0)
	Northern Africa	223.8 (167.6–293.6)	136.6 (109.3–174.3)
	Western Africa	5.8 (1.7–13.0)	19.0 (13.5–29.9)
	Eastern Africa	3.5 (0.7–9.3)	2.3 (1.1–4.9)
	Middle Africa	0.1 (0.1–0.2)	0.6 (0.5–0.9)
Asia		223.5 (164.9–282.8)	208.7 (181.1–235.7)
	Western Asia	193.9 (148.3–236.7)	154.8 (138.7–168.9)
	Southern Asia	24.9 (14.9–38.3)	48.3 (40.0–57.6)
	Central Asia	4.7 (1.8–7.9)	5.7 (2.4–9.2)

* These geographic regions are based on continental regions, which are further subdivided into subregions based on the United Nations “Standard Country or Area Codes for Statistical Use” (https://unstats.un.org/unsd/methodology/m49/) (accessed on 1 July 2023). ^#^ The mean (2.5–97.5% percentiles) was calculated for the estimation. TOSV, Toscana virus.

## Data Availability

The datasets used and/or analyzed during the current study are available from the corresponding author upon reasonable request.

## Supplementary Materials

The following supporting information can be downloaded at https://www.mdpi.com/article/10.3390/v17010015/s1,Text S1: Data collection and management; Text S2: Model building; Figure S1: The documented spatial distribution of the two sandfly vectors of TOSV; Figure S2: ROC curves and AUC values of the BRT; Figure S3: Probability of occurrence of *P. perniciosus* and relative contributions (RCs) of the significant predictors (RC > 5%) for *P. perniciosus* based on BRT models; Figure S4: Probability of occurrence of *P. perfiliewi* and relative contributions (RCs) of the significant predictors (RC > 5%) for *P. perfiliewi* based on BRT models; Table S1: The inclusion and exclusion criteria for screening publications; Table S2: The laboratory tests used to detect TOSV infections in the included studies; Table S3: Variables used for ecological modeling in this study; Table S4: Original resolutions and extents of source datasets; Table S5: The specific references for TOSV and sandflies.
