# Supplementary material for: Epidemiology and Ecology of Toscana Virus Infection and Its Global Risk Distribution
Source: Viruses. 2024 Dec 25;17(1):15. doi: 10.3390/v17010015 (PMC11768567; doi:10.3390/v17010015)
Supplement: Supplementary file 1 [file viruses-17-00015-s001.zip › viruses-3365759-supplementary.pdf]

Appendix A

Supplement to: **Epidemiology and ecology of Toscana virus infection and its global risk distribution**

Contents

Contents 1
Supplementary Methods 2
Text S1: Data collection and management 2
Extracting data related to TOSV from publications 2
Extracting data related to vectors from publications 2
Geo-positioning of the occurrence data 2
Assembling occurrence data and covariates 3
Clinical spectrum of TOSV infections 3
Text S2: Model building 4
Supplementary References 1 5
Supplementary Figures 6
Figure S1 The documented spatial distribution of the two sandfly vectors of TOSV. 6
Figure S2 ROC curves and AUC values of the BRT. 7
Figure S3 Probability of occurrence of *P. perniciosus* and relative contributions (RCs) of the significant predictors (RC >5%) for *P. perniciosus* based on BRT models. 8
Figure S4 Probability of occurrence of *P. perfiliewi* and relative contributions (RCs) of the significant predictors (RC >5%) for *P. perfiliewi* based on BRT models. 9
Supplementary Tables 10
Table S1 The inclusion and exclusion criteria for screening publications. 10
Table S2 The laboratory tests used to detect TOSV infections in the included studies. 11
Table S3 Variables used for ecological modelling in this study. 12
Supplementary References 2 14
Table S4 Original resolutions and extents of source datasets. 15
Table S5 The specific references for TOSV and sandflies. 17
Supplementary References 3 18

Supplementary Methods

Text S1: Data collection and management

Extracting data related to TOSV from publications

The following data were extracted from each selected article: article title, authors, publication year, study period (sample collection time), study site (up to the highest resolution), latitude and longitude of the site, detection method, type of host (sandfly, vertebrate or human being), genotype, the total number of tested samples, and the number of positive samples.

Extracting data related to vectors from publications

The following data were extracted from each selected article: article title, authors, publication year, study period (sample collection time), study site (up to the highest resolution), latitude and longitude of the site, identification method, type of species.

Geo-positioning of the occurrence data

To geocode occurrences of TOSV, an occurrence is defined as one or more confirmed infection(s) with TOSV at a unique location (geocoordinates, polygons, or 10 km×10 km pixels) during any period, regardless of the type of the host or the time of detection. For the sandfly vectors, an occurrence is defined as the identification of a specific vector species at a unique location (geocoordinates, polygons, or 10 km×10 km pixels) during any period. Serological tests in humans were excluded when modeling for TOSV due to potential cross-reactivity among *phleboviruses* (Additional file 1: Table S2) [1,2]. Whenever available, we extracted geocoordinates from peer-reviewed articles reporting TOSV or vector occurrences as "point" data. When point information was not available, we extracted the location as a two-dimensional bounded region, or a "polygon". A polygon is usually an administrative unit, such as a county, a city, or a province. Occasionally, it could be a customized sampling region. For each polygon, the coordinates of its geographic centroid were queried from Google Maps. All location data were geopositioned with the highest possible precision and checked to ensure coordinates were accurate and duplicates were removed, so that each individual record used in our model represents a unique occurrence of TOSV or vector. After that geocoordinates (latitude and longitude) of sites were second queried using Google Maps to make sure they match the locations mentioned in the articles as well as to remove duplicated sites. All occurrence data underwent quality control to ensure reliability and precision of geo- positioning. Specifically, all occurrence data were double checked by two investigators (X-GH, Q-MZ) independently, with special attention to the TOSV species and their

sampling times and locations. In addition, names of study sites were updated if current names slightly differ from the ones used in the articles. In addition, classification of locations of occurrence as "Points" or "Polygons" were cross- checked by the two investigators.

Assembling occurrence data and covariates

We created a global grid-map with a resolution of 10 km×10 km using ArcGIS 10.7 (Esri Inc, Redlands, CA, USA) and then associated each grid with ecological variables. Each occurrence was matched to the grid-map according to its coordinate. For polygon-type occurrence records, we assigned the grid containing the centroid of the polygon as the occurrence grid. Only one occurrence was counted if multiple records were associated with the same grid. For ecological modeling, we need to associate ecological variables with each grid (occurrence or non-occurrence). The average of each ecological variable over its corresponding time span was calculated for each grid [3]. If the original occurrence record is a point location, association of point data with grids is straightforward and no more processing is needed. If the original occurrence record is a polygon, the assigned occurrence grid may not be the true location, and the ecological variables associated with that grid may not represent the true ecological condition for that occurrence. To minimize potential ecological fallacy, we first exclude all polygon occurrence records with an area larger than 20 km × 20 km from ecological modeling because of insufficient resolution. For polygon occurrence records with an area no larger than 20 km × 20 km, we calculated the mean of each ecological variable across all grids within the polygon and associated the mean value with the occurrence grid, i.e., the grid containing the centroid of the polygon. All occurrence grids were considered as "presence". For each occurrence grid we sample pseudo-absence grids as "absence" with a case-to-control ratio of 1:3 for the modelling analysis [4,5]. For each occurrence grid, the sampling was restricted to beyond 200 km from the center point of the occurrence grid and within the entire study area (latitude 10°N–65°N, longitude 20°W–70°E).

Clinical spectrum of TOSV infections

When calculating the characteristic information, infections with incomplete characteristic information were excluded (e.g., fever or headache, without mentioning the presence or absence of other symptoms), and only included the infections that reported the symptoms completely. The different symptoms were categorized and the frequency and symptom proportion of each symptom were summarized.

Text S2: Model building

Due to the strong intercorrelation among climatic variables, we used the "caret" package to examine the correlation between all climatic variables (BIO1-19) in R 4.1.2. Variables with correlation coefficients greater than 0.80 were excluded from our analysis [3]. The modeling uses the Boosted Regression Trees (BRT)method, which is a machine learning technique for regression and classification. To control the number of variables involved in the final modeling, we first fitted an initial model for TOSV or sandflies. Predictors with relative contributions (RCs) greater than 2% (for TOSV) or 3% (for sandflies) were retained for the formal model-building process. In the final model, we randomly divided the data into an 80% training set and a 20% test set and fitted a BRT model, which was repeated 100 times [3,6]. That is, we obtained 100 models based on the 100 training datasets for each target species, to which we refer as a model assembly. Using these presence and pseudo-absence locations and ecological predictors, BRT models were fitted using the "gbm.step" function in "dismo" package in R 4.1.2 (R Foundation for Statistical Computing, Vienna, Austria) with a tree complexity of five, a learning rate of 0·005, and a bagging fraction of 75% based on their satisfactory performance in our previous research [3,6,7]. A 10-fold cross validation was used to identify the optimal number of trees using the gbm.step function in the R package "dismo". Due to both the data size (41 predictors for TOSV and 39 predictors for sandflies) and the number of models runs ([TOSV and 2 sandfly species] × 100), we did not perform a full cross-validation optimization for all model configuration parameters. However, we performed a sensitivity analysis using a learning rate of 0.01 for TOSV and sandflies but found no substantial difference in the contribution estimates.

Supplementary References 1

[1] Ergünay K, Litzba N, Lo MM, et al. Performance of various commercial assays for the detection of Toscana virus antibodies. Vector Borne Zoonotic Dis. 2011 Jun;11(6):781-7.

[2] Pierro A, Ficarelli S, Ayhan N, et al. Characterization of antibody response in neuroinvasive infection caused by Toscana virus. Clin Microbiol Infect. 2017 Nov;23(11):868-873.

[3] Che TL, Jiang BG, Xu Q, et al. Mapping the risk distribution of Borrelia burgdorferi sensu lato in China from 1986 to 2020: a geospatial modelling analysis. Emerg Microbes Infect. 2022 Dec;11(1):1215-1226.

[4] VanDerWal J, Shoo LP, Graham C, et al. Selecting pseudo-absence data for presence-only distribution modeling: How far should you stray from what you know? Ecological Modelling. 2009 2009/02/24/;220(4):589-594.

[5] Barbet-Massin M, Jiguet F, Albert C, et al. Selecting Pseudo-Absences for Species Distribution Models: How, Where and How Many? Methods in Ecology and Evolution. 2012 04/01;3:327–338.

[6] Zhang YY, Sun YQ, Chen JJ, et al. Mapping the global distribution of spotted fever group rickettsiae: a systematic review with modelling analysis. Lancet Digit Health. 2023 Jan;5(1):e5-e15.

[7] Zhao GP, Wang YX, Fan ZW, et al. Mapping ticks and tick-borne pathogens in China. Nat Commun. 2021 Feb 17;12(1):1075.

Supplementary Figures

Figure S1 The documented spatial distribution of the two sandfly vectors of TOSV.

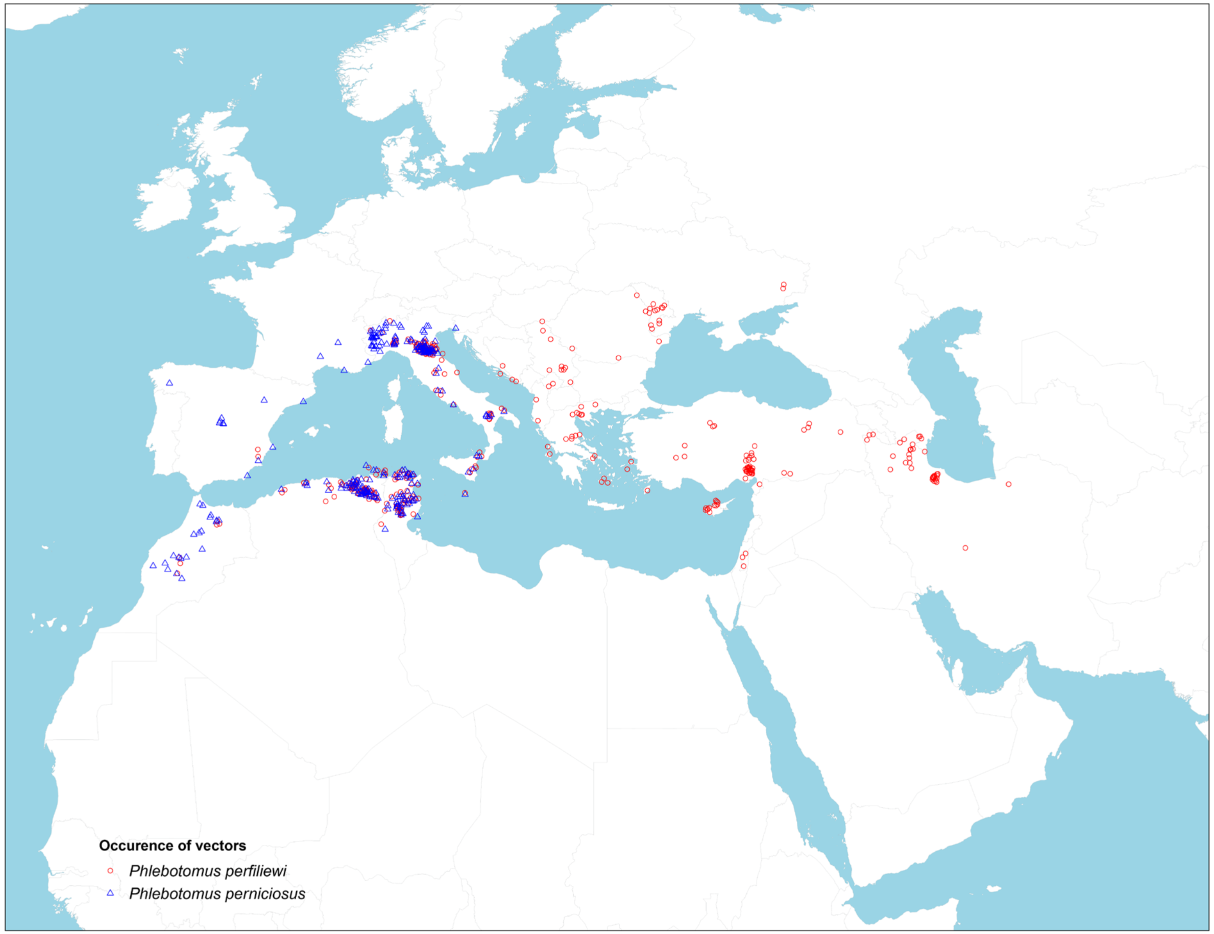

Figure S2 ROC curves and AUC values of the BRT.

(a) ROC curves and AUC values of *P. perniciosus* models. **(b)** ROC curves and AUC values of *P. perfiliewi* models. **(c)** ROC curves and AUC values of TOSV infection models.

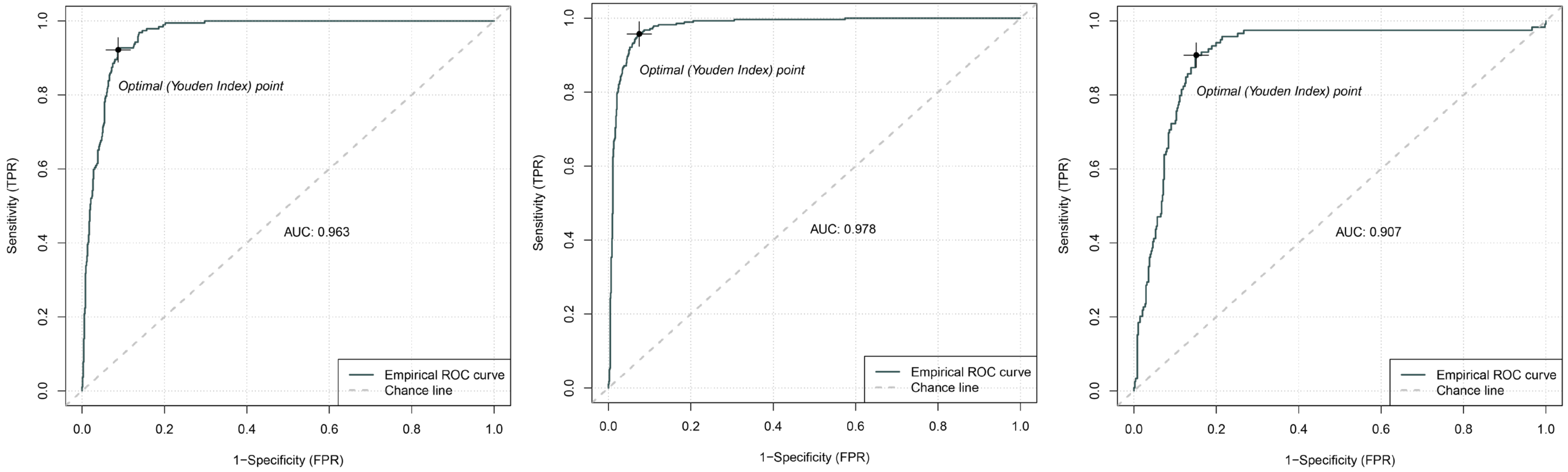

a b c

Figure S3 Probability of occurrence of *P. perniciosus* and relative contributions (RCs) of the significant predictors (RC >5%) for *P. perniciosus* based on BRT models.

Panel a, Maps displaying probability data (latitude 10°N–65°N, longitude 20°W–70°E). **Panel b,** RCs of predictors on occurrence probability for *P. perniciosus*. **Panel c,** Response curve of predictors in BRT models for *P. perniciosus*. Mean curves (red) and 95% percentiles (grey) show the contribution on predicting the probability of occurrence. The histograms show the frequency distributions of the predictors.

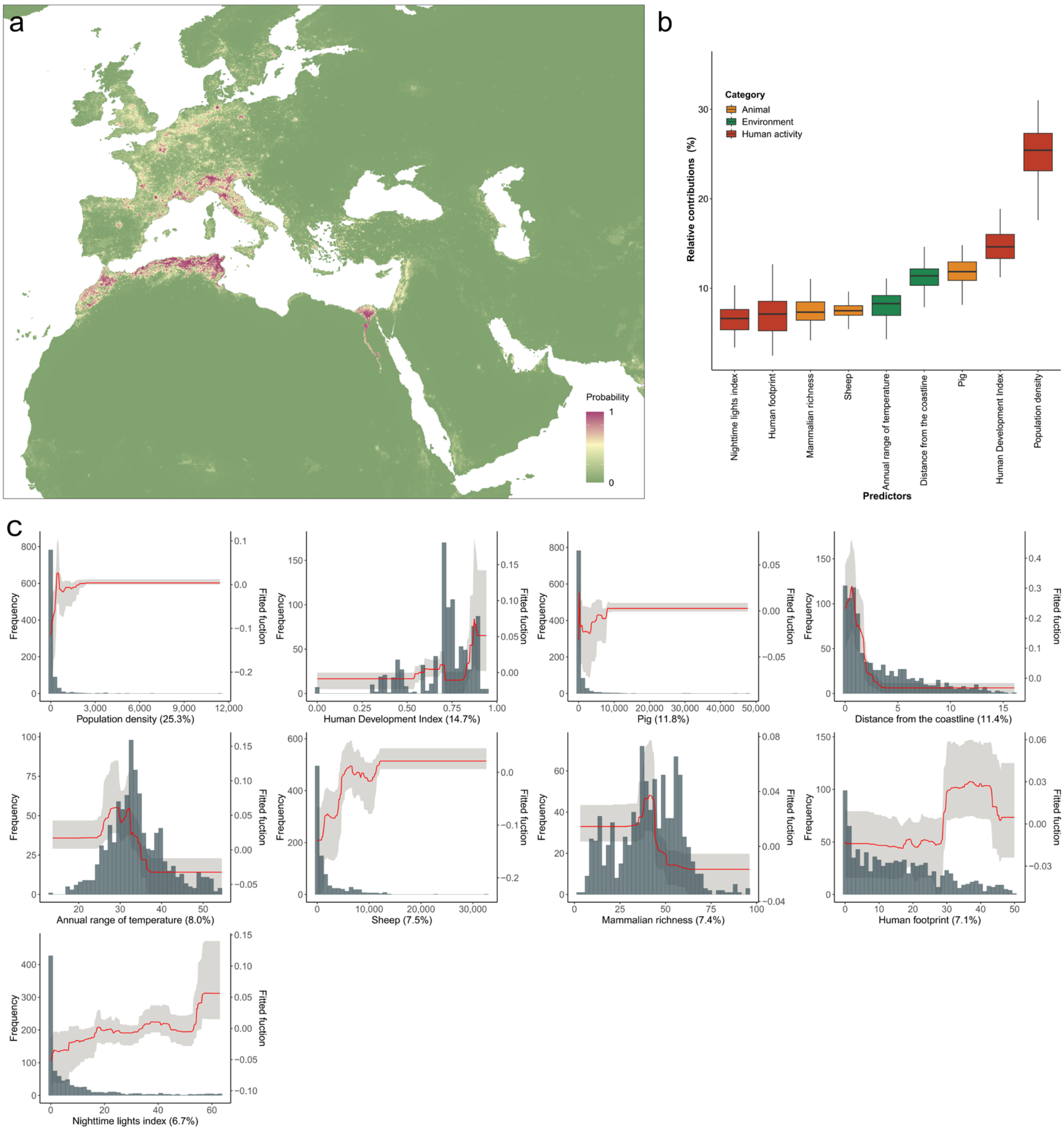

Figure S4 Probability of occurrence of *P. perfiliewi* and relative contributions (RCs) of the significant predictors (RC >5%) for *P. perfiliewi* based on BRT models.

Panel a, Maps displaying probability data (latitude 10°N–65°N, longitude 20°W–70°E). **Panel b,** RCs of predictors on occurrence probability for *P. perfiliewi*. **Panel c,** Response curve of predictors in BRT models for *P. perfiliewi*. Mean curves (red) and 95% percentiles (grey) show the contribution on predicting the probability of occurrence. The histograms show the frequency distributions of the predictors.

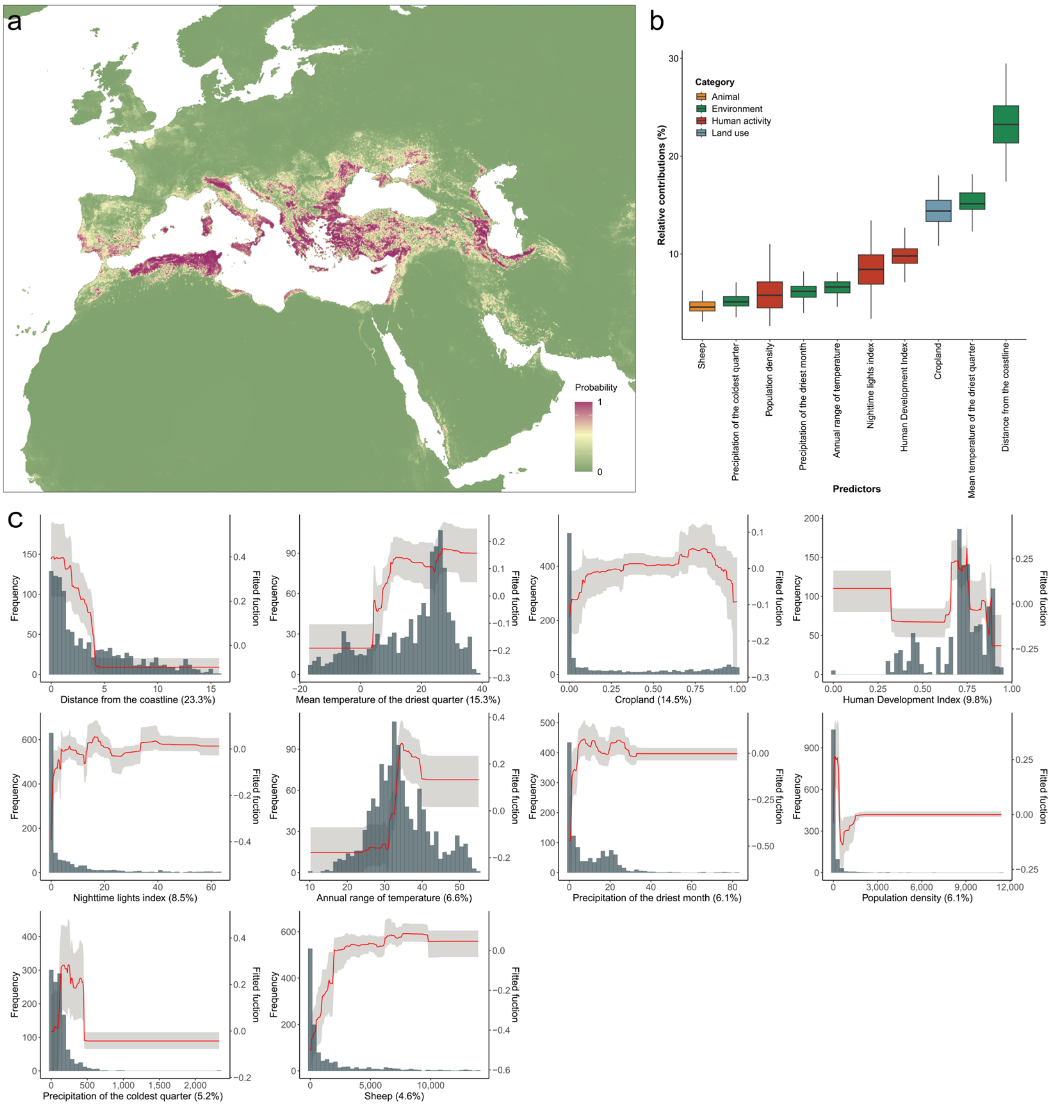

Supplementary Tables

Table S1 The inclusion and exclusion criteria for screening publications.

Criteria	Guidance	Outcome
**Title/Abstract screening**		
#1: Pathogens	Does the Title/Abstract refer the pathogens which belong to TOSV?	If Yes, remain and evaluate #2. If No, exclude.
#2: Source of infection	Does the Title/Abstract refer the pathogens which are from natural environment?	If Yes, remain and evaluate #3. If No, exclude.
#3: Tested objects	Does the Title/Abstract refer the pathogens which are detected from vectors, hosts, or humans?	If Yes, remain and evaluate #4. If No, exclude.
#4: Not review	Does the Title/Abstract refer to the article which is Not a review? (Not reviewing the published articles, with presenting new primary data)	If No, remain for full-text review. If Yes, exclude.
**Full text screening**		
#1: Re-screening	Does the article meet the screening criteria before?<br>1-pathogens belong to TOSV?<br>2-infection in the natural environment<br>3-not drug or vaccine trials<br>4-not molecular research of TOSV<br>5-detected from vectors, hosts, or humans	If Yes, remain and evaluate #2. If No, exclude.
#2: Laboratory method	Does the article refer the specific detection methods?<br>1-detailed specimen used for testing (e.g. vectors or blood from hosts or humans)<br>2-pathogen-based testing method (e.g. serological or molecular)<br>3-TOSV identified in the detection	If Yes, remain and evaluate #3. If No, exclude.
#3: Geographical information	Does the article refer the geographical information?<br>1-geographic location information at country or subnational administrative divisions levels<br>2-exact locations or only marked the latitude and longitude<br>3-explicit locations of getting infections when imported	If Yes, remain for data extracting. If No, exclude.

Table S2 The laboratory tests used to detect TOSV infections in the included studies.

Category	Detection methods
Infections in vectors	(1)/ (2)
Infections in vertebrates	(1)/ (2)/ (3)/ (4)
*Confirmed human infections	(1)/ (2)/ (3)/ (4)/ (5)
Serological positive in humans	Serological positives as those testing antibody positive, excluding situations encompassed within the definition of confirmed human infections.

*The definition of "confirmed human infections" is referenced from the 2015 case definition of arboviral diseases available on the website of the Centers for Disease Control and Prevention (CDC): https://ndc.services.cdc.gov/case-definitions/arboviral-diseases-neuroinvasive-and-non-neuroinvasive-2015/.
(1) Molecular detection and sequence determination;
(2) Isolation and cultivation of pathogens from samples;
(3) Virus-specific IgM antibodies in CSF and a negative result for other IgM antibodies in CSF for arboviruses known to be endemic in the region where exposure occurred;
(4) Virus-specific antibodies in serum with confirmatory virus-specific neutralizing antibodies in the same or a later specimen;
(5) Four-fold or greater change in virus-specific quantitative antibody titers in paired sera, or a seroconversion of specific antibodies.

Table S3 Variables used for ecological modelling in this study.

Category	Data	Variable	Description	Reference
Animal	Livestock	Buffalo	Density of buffalo (heads per km^2)	[1-4]
		Cattle	Density of cattle (heads per km^2)	
		Goat	Density of goat (heads per km^2)	
		Horse	Density of horse (heads per km^2)	
		Pig	Density of pig (heads per km^2)	
		Sheep	Density of sheep (heads per km^2)	
	Mammals*	Mammalian richness	The number of mammal species per km^2	
Environment	Climate	BIO1	Annual mean temperature (°C)	[4-10]
		BIO2*	Mean diurnal range (Mean of monthly (max temp-min temp)) (°C)	
		BIO3	Isothermality (BIO2/ BIO7) (*100)	
		BIO4	Temperature seasonality (standard deviation*100)	
		BIO5	Max temperature of warmest month (°C)	
		BIO6	Min temperature of coldest month (°C)	
		BIO7*	Annual range of temperature (BIO5- BIO6) (°C)	
		BIO8*	Mean temperature of wettest quarter (°C)	
		BIO9*	Mean temperature of driest quarter (°C)	
		BIO10	Mean temperature of warmest quarter (°C)	
		BIO11	Mean temperature of coldest quarter (°C)	
		BIO12	Annual precipitation (mm)	
		BIO13*	Precipitation of wettest month (mm)	
		BIO14*	Precipitation of driest month (mm)	
		BIO15*	Precipitation seasonality (Coefficient of variation)	
		BIO16	Precipitation of wettest quarter (mm)	
		BIO17	Precipitation of driest quarter (mm)	
		BIO18	Precipitation of warmest quarter (mm)	
		BIO19*	Precipitation of coldest quarter (mm)	
	Leaf area index	Leaf area index	Area of leaves (m^2) over a unit of land (m^2)	
	Elevation	Elevation	Average elevation (m)	

	Coastline	Distance from coastline	Average distance (km)	
Land use	Land use	Cropland	Percentage coverage of cropland (%)	[4, 8-10]
		Mixed cropland and nature vegetation	Percentage coverage of mixed cropland and nature vegetation (%)	
		Forest	Percentage coverage of forest (%)	
		Shrubland	Percentage coverage of shrubland (%)	
		Mixed tree, shrub, and herbaceous	Percentage coverage of mixed tree, shrub and herbaceous (%)	
		Grassland	Percentage coverage of grassland (%)	
		Lichens and mosses	Percentage coverage of lichens and mosses (%)	
		Sparse vegetation land	Percentage coverage of sparse vegetation land (%)	
		Flooded vegetation	Percentage coverage of flooded vegetation (%)	
		Urban construction land	Percentage coverage of urban construction land (%)	
		Bare areas	Percentage coverage of bare areas (%)	
		Water body	Percentage coverage of inland water body (%)	
		Ice and snow	Percentage coverage of ice and snow (%)	
Human activity	Human activity	Global downscaled GDP	The assessment of GDP per raster	[4]
		Population density	Average population count (count per km²)	
		Nighttime lights index	Average nighttime lights index (per 10 km²)	
		Time to healthcare by motorized transportation	Travel time to healthcare facilities with motorized transportation (hours per 5 km²)	
		Time to healthcare by walking	Travel time to healthcare facilities without motorized transportation (hours per 5 km²)	
		Human Development Index	Human Development Index (per 10 km²)	
		Gini coefficient	Gini coefficient (per 10 km²)	
		Human Footprint	The annual dynamics of the global human footprint per km²	

In order to mitigate the issue of multicollinearity, a screening process was applied to the BIO (1–19) variables. Those variables that were marked with an asterisk (*) were subsequently chosen and incorporated into the construction of the model

Supplementary References 2

[1] Verani P, Ciufolini MG, Caciolli S, et al. Ecology of viruses isolated from sand flies in Italy and characterized of a new Phlebovirus (Arabia virus). Am J Trop Med Hyg. 1988 Mar;38(2):433-9.

[2] Ayhan N, Sherifi K, Taraku A, et al. High Rates of Neutralizing Antibodies to Toscana and Sandfly Fever Sicilian Viruses in Livestock, Kosovo. Emerg Infect Dis. 2017 Jun;23(6):989-992.

[3] Fezaa O, M'Ghirbi Y, Savellini GG, et al. Serological and molecular detection of Toscana and other Phleboviruses in patients and sandflies in Tunisia. BMC Infect Dis. 2014 Nov 15;14:598.

[4] Risueño J, Muñoz C, Pérez-Cutillas P, et al. Understanding Phlebotomus perniciosus abundance in south-east Spain: assessing the role of environmental and anthropic factors. Parasit Vectors. 2017 Apr 19;10(1):189.

[5] Gálvez R, Descalzo MA, Miró G, et al. Seasonal trends and spatial relations between environmental/meteorological factors and leishmaniosis sand fly vector abundances in Central Spain. Acta Trop. 2010 Jul-Aug;115(1-2):95-102.

[6] Cecílio P, Cordeiro-da-Silva A, Oliveira F. Sand flies: Basic information on the vectors of leishmaniasis and their interactions with Leishmania parasites. Commun Biol. 2022 Apr 4;5(1):305.

[7] Talbi FZ, El Ouali Lalami A, Fadil M, et al. Entomological Investigations, Seasonal Fluctuations and Impact of Bioclimate Factors of Phlebotomines Sand Flies (Diptera: Psychodidae) of an Emerging Focus of Cutaneous Leishmaniasis in Aichoun, Central Morocco. J Parasitol Res. 2020;2020:6495108.

[8] Alcover MM, Ballart C, Martín-Sánchez J, et al. Factors influencing the presence of sand flies in Majorca (Balearic Islands, Spain) with special reference to Phlebotomus pernicious, vector of Leishmania infantum. Parasit Vectors. 2014 Sep 4;7:421.

[9] Tsirigotakis N, Pavlou C, Christodoulou V, et al. Phlebotomine sand flies (Diptera: Psychodidae) in the Greek Aegean Islands: ecological approaches. Parasit Vectors. 2018 Feb 20;11(1):97.

[10] Calzolari M, Romeo G, Munari M, et al. Sand Flies and Pathogens in the Lowlands of Emilia-Romagna (Northern Italy). Viruses. 2022 Oct 7;14(10).

Table S4 Original resolutions and extents of source datasets.

Variable	Spatial resolution	Temporal extent	Source of data	Website	Reference
Climate data	0°2.5′	1980-2018*	WorldClim	https://www.worldclim.org/	Fick SE, Hijmans RJ. WorldClim 2: new 1-km spatial resolution climate surfaces for global land areas. Int. J. Climatol., 2017; 37: 4302-15.<br>Harris I, Jones PD, Osborn TJ, Lister DH. Updated high-resolution grids of monthly climatic observations – the CRU TS3.10 Dataset. Int. J. Climatol., 2014; 34: 623-42.
Leaf area index	8 km	1981-2019	Resource and Environment Science and Data Center	https://www.resdc.cn/	Yang L, Liu R, Chen JM. Retrospective retrieval of long-term consistent global leaf area index (1981-2011) from combined AVHRR and MODIS data. J Geophys Res Biogeosci, 2015; 117.
Land cover	0.3 km	1992-2019	European Space Agency	https://maps.elie.ucl.ac.be/CCI/	European Space Agency. ESA Land Cover Climate Change Initiative (Land_Cover_cci): Global Land Cover Maps, Version 2.0.7. https://catalogue.ceda.ac.uk/uuid/b382ebe6679d44b8b0e68ea4ef4b701c/ (accessed May 28, 2021).
Elevation	1 km	2010	EarthEnv (DEM90)	http://www.earthenv.org/	Robinson N, Regetz J, Guralnick RP. EarthEnv-DEM90: A nearly-global, void-free, multi-scale smoothed, 90m digital elevation model from fused ASTER and SRTM data. ISPRS, 2014; 87: 57-67.
Distance from coastline	1 km	2012	Glaciology and Geocryology Data Center, National Earth System Science Data Sharing Infrastructure, National Science & Technology Infrastructure of China	http://westdc.geodata.cn/index.html	Glaciology and Geocryology Data Center, National Earth System Science Data Sharing Infrastructure, National Science & Technology Infrastructure of China (http://www. http://westdc.geodata.cn/index.html).
Livestock density	1 km	2010	Food and Agriculture Organization (FAO)	http://www.fao.org/livestock-systems/en/	Gilbert M, Nicolas G, Cinardi G, et al. Global distribution data for cattle, buffaloes, horses, sheep, goats, pigs, chickens and ducks in 2010. Sci Data, 2018; 5: 180227.
Mammalian richness	0°0′30″	2013	International Union for Conservation of Nature (IUCN)	https://sedac.ciesin.columbia.edu/data/collection/species	International Union for Conservation of Nature - IUCN, and Center for International Earth Science Information Network - CIESIN - Columbia University. 2015. Gridded Species Distribution: Global Mammal Richness Grids, 2015 Release. Palisades, NY: NASA Socioeconomic Data and Applications Center (SEDAC).
Population number	1 km	2020	WorldPop 2020	https://www.worldpop.org/	WorldPop. Population counts, unconstrained global mosaics 2000-2020 (1 km resolution), 2020. https://www.worldpop.org/geodata/listing?id=64/ (accessed Apr 12, 2021).
Nighttime lights index	0°0′30″	1992-2013	the U.S. Air Force Defense Meteorological Satellite Program (DMSP)	https://eogdata.mines.edu/products/dmsp/#v4	Elvidge CD, Baugh KE, Kihn EA, Kroehl HW, & Davis ER. Mapping city lights with nighttime data from the DMSP Operational Linescan System. Photogrammetric Engineering and Remote Sensing., 1997; 63: 727-734.<br>Baugh K, Elvidge CD, Ghosh T, & Ziskin D. Development of a 2009 stable lights product using DMSP-OLS data. Proceedings of the Asia-Pacific Advanced Network., 2010; 30: 114.
Global Downscaled GDP	0°15′	1990, 2025	NASA Socioeconomic Data and Applications Center (SEDAC)	https://sedac.ciesin.columbia.edu/	Gaffin SR, X Xing, & G Yetman. Country-Level GDP and Downscaled Projections Based on the SRES A1, A2, B1, and B2 Marker Scenarios, 1990-2100. Palisades, New York: NASA Socioeconomic Data and Applications Center (SEDAC). 2002.<br>Gaffin SR, C Rosenzweig, X Xing, & G Yetman. Downscaling and Geo-spatial Gridding of Socio-economic Projections from the IPCC Special Report on Emissions Scenarios (SRES). Global Environmental Change., 2004; 14: 105-123.

Travel time to healthcare facilities	5 km	2000-2020	Malaria Atlas Project	https://malariaatlas.org/	Weiss DJ, Nelson A, Vargas-Ruiz CA, et al. Global maps of travel time to healthcare facilities. Nat Med., 2020; 26: 1835-1838.
Human Development Index (HDI)	0°5′	1990–2015	Aalto University	https://datadryad.org/stash/dataset/doi:10.5061/dryad.dk1j0	Kummu M, Taka M, & Guillaume J. Gridded global datasets for Gross Domestic Product and Human Development Index over 1990–2015. Sci Data, 2018; 180004.
Gini coefficient	1°	2010	Zenodo	https://zenodo.org/record/4635734	Mirza MU, Xu C, Bavel BV, van Nes EH, & Scheffer M. Global inequality remotely sensed. Proc Natl Acad Sci U S A, 2021; 118:e1919913118.
Human Footprint	1 km	2000–2018	Scientific Data	https://www.gisrsdata.com/	Mu, Haowei; Li, Xuecao; Wen, Yanan; Huang, Jianxi; Du, Peijun; Su, Wei; et al. (2021): An annual global terrestrial Human Footprint dataset from 2000 to 2018. figshare. Figure. https://doi.org/10.6084/m9.figshare.16571064.v5

*This dataset is recalculated according to historical monthly weather data between 1980 to 2018, which is the main period when the TOSV was detected, using the 'biovars' function in the R package dismo.

Table S5 The specific references for TOSV and sandflies.

**Category**	Reference
TOSV	[1-199]
P.perniciosus and *P.perfiliewi*	[2, 22, 86, 88, 101, 102, 106, 111, 130, 138, 147, 148, 164, 173, 175-177, 200-372]

Supplementary References 3

[1] Alkan C, Allal-Ikhlef AB, Alwassouf S, et al. Virus isolation, genetic characterization and seroprevalence of Toscana virus in Algeria. Clin Microbiol Infect. 2015 Nov;21(11):1040.e1-9.

[2] Remoli ME, Fortuna C, Marchi A, et al. Viral isolates of a novel putative phlebovirus in the Marche Region of Italy. Am J Trop Med Hyg. 2014 Apr;90(4):760-3.

[3] de Ory F, Avellón A, Echevarría JE, et al. Viral infections of the central nervous system in Spain: a prospective study. J Med Virol. 2013 Mar;85(3):554-62.

[4] McGill F, Tokarz R, Thomson EC, et al. Viral capture sequencing detects unexpected viruses in the cerebrospinal fluid of adults with meningitis. J Infect. 2022 Apr;84(4):499-510.

[5] Ergunay K, Kaplan B, Okar S, et al. Urinary detection of toscana virus nucleic acids in neuroinvasive infections. J Clin Virol. 2015 Sep;70:89-92.

[6] Sanbonmatsu-Gámez S, Pedrosa-Corral I, Navarro-Marí JM, et al. Update in Diagnostics of Toscana Virus Infection in a Hyperendemic Region (Southern Spain). Viruses. 2021 Jul 23;13(8).

[7] Baldelli F, Ciufolini MG, Francisci D, et al. Unusual presentation of life-threatening Toscana virus meningoencephalitis. Clin Infect Dis. 2004 Feb 15;38(4):515-20.

[8] Sanbonmatsu-Gámez S, Pérez-Ruiz M, Palop-Borrás B, et al. Unusual manifestation of toscana virus infection, Spain. Emerg Infect Dis. 2009 Feb;15(2):347-8.

[9] Quattrone F, Mazzetti P, Aquino F, et al. Two clusters of Toscana virus meningo-encephalitis in Livorno Province and Elba Island, July-September 2018. Ann Ig. 2020 Nov-Dec;32(6):674-681.

[10] Schwarz TF, Gilch S, Jäger G. Travel-related Toscana virus infection. Lancet. 1993 Sep 25;342(8874):803-4.

[11] Amaro F, Zé-Zé L, Luz MT, et al. Toscana Virus: Ten Years of Diagnostics in Portugal. Acta Med Port. 2021 Oct 1;34(10):677-681.

[12] De Ory F, Gegúndez MI, Fedele CG, et al. [Toscana virus, West Nile virus and lymphochoriomeningitis virus as causing agents of aseptic meningitis in Spain]. Med Clin (Barc). 2009 Apr 25;132(15):587-90.

[13] Charrel RN, Izri A, Temmam S, et al. Toscana virus RNA in Sergentomyia minuta files. Emerg Infect Dis. 2006 Aug;12(8):1299-300.

[14] Mosnier E, Charrel R, Vidal B, et al. Toscana virus myositis and fasciitis. Med Mal Infect. 2013 May;43(5):208-10.

[15] Wenzel M, Afzali AM, Hoffmann D, et al. Toscana Virus Meningoencephalitis in Upper Bavaria. Dtsch Arztebl Int. 2022 Aug 8;119(31-32):546-547.

[16] Veater J, Mehedi F, Cheung CK, et al. Toscana virus meningo-encephalitis: an important differential diagnosis for elderly travellers returning from Mediterranean countries. BMC Geriatr. 2017 Aug 29;17(1):193.

[17] Chagneau CV, Mansuy J-M, Barthe C, et al. Toscana virus meningitis in Southwestern France. J Clin Virol. 2016;82.

[18] Santos L, Simões J, Costa R, et al. Toscana virus meningitis in Portugal, 2002-2005. Euro Surveill. 2007 Jun 1;12(6):E3-4.

[19] Jiménez BC, Fuentes ME, Sánchez-Seco MP, et al. [Toscana virus meningitis in an Argentinian immigrant]. Enferm Infecc Microbiol Clin. 2012 May;30(5):272-3.

[20] Karunaratne K, Davies N. Toscana virus meningitis following a holiday in Elba, Italy. Br J Hosp Med (Lond). 2018 May 2;79(5):292.

[21] Cordey S, Bel M, Petty TJ, et al. Toscana virus meningitis case in Switzerland: an example of the ezVIR bioinformatics pipeline utility for the identification of emerging viruses. Clin Microbiol Infect. 2015 Apr;21(4):387.e1-4.

[22] Bichaud L, Dachraoui K, Piorkowski G, et al. Toscana virus isolated from sandflies, Tunisia. Emerg Infect Dis. 2013 Feb;19(2):322-4.

[23] Es-sette N, Ajaoud M, Anga L, et al. Toscana virus isolated from sandflies, Morocco. Parasit Vectors. 2015 Apr 3;8:205.

[24] Dupouey J, Bichaud L, Ninove L, et al. Toscana virus infections: a case series from France. J Infect. 2014 Mar;68(3):290-5.

[25] Braito A, Corbisiero R, Corradini S, et al. Toscana virus infections of the central nervous system in children: a report of 14 cases. J Pediatr. 1998 Jan;132(1):144-8.

[26] Vocale C, Bartoletti M, Rossini G, et al. Toscana virus infections in northern Italy: laboratory and clinical evaluation. Vector Borne Zoonotic Dis. 2012 Jun;12(6):526-9.

[27] Calisher CH, Weinberg AN, Muth DJ, et al. Toscana virus infection in United States citizen returning from Italy. Lancet. 1987 Jan 17;1(8525):165-6.

[28] Dobler G, Treib J, Haass A, et al. Toscana virus infection in German travellers returning from the Mediterranean. Infection. 1997 Sep-Oct;25(5):325.

[29] Cardeñosa N, Kaptoul D, Fernández-Viladrich P, et al. Toscana virus infection in Catalonia (Spain). Vector Borne Zoonotic Dis. 2013 Apr;13(4):273-5.

[30] Kay MK, Gibney KB, Riedo FX, et al. Toscana virus infection in American traveler returning from Sicily, 2009. Emerg Infect Dis. 2010 Sep;16(9):1498-500.

[31] Gabriel M, Resch C, Günther S, et al. Toscana virus infection imported from Elba into Switzerland. Emerg Infect Dis. 2010 Jun;16(6):1034-6.

[32] Amaro F, Luz T, Parreira P, et al. [Toscana virus in the Portuguese population: serosurvey and clinical cases]. Acta Med Port. 2011 Dec;24 Suppl 2:503-8.

[33] Sanbonmatsu-Gámez S, Pérez-Ruiz M, Collao X, et al. Toscana virus in Spain. Emerg Infect Dis. 2005 Nov;11(11):1701-7.

[34] Magurano F, Baggieri M, Gattuso G, et al. Toscana virus genome stability: data from a meningoencephalitis case in Mantua, Italy. Vector Borne Zoonotic Dis. 2014 Dec;14(12):866-9.

[35] Dersch R, Sophocleous A, Cadar D, et al. Toscana virus encephalitis in Southwest Germany: a retrospective study. BMC Neurol. 2021 Dec 22;21(1):495.

[36] Howell BA, Azar MM, Landry ML, et al. Toscana virus encephalitis in a traveler returning to the United States. J Clin Microbiol. 2015 Apr;53(4):1445-7.

[37] Osborne JC, Khatamzas E, Misbahuddin A, et al. Toscana virus encephalitis following a holiday in Sicily. Pract Neurol. 2016 Apr;16(2):139-41.

[38] Di Nicuolo G, Pagliano P, Battisti S, et al. Toscana virus central nervous system infections in southern Italy. J Clin Microbiol. 2005 Dec;43(12):6186-8.

[39] Kuhn J, Bewermeyer H, Hartmann-Klosterkoetter U, et al. Toscana virus causing severe meningoencephalitis in an elderly traveller. J Neurol Neurosurg Psychiatry. 2005 Nov;76(11):1605-6.

[40] Okar SV, Bekircan-Kurt CE, Hacıoğlu S, et al. Toscana virus associated with Guillain-Barré syndrome: a case-control study. Acta Neurol Belg. 2021 Jun;121(3):661-668.

[41] Peyrefitte CN, Devetakov I, Pastorino B, et al. Toscana virus and acute meningitis, France. Emerg Infect Dis. 2005 May;11(5):778-80.

[42] Ergunay K, Aydogan S, Ilhami Ozcebe O, et al. Toscana virus (TOSV) exposure is confirmed in blood donors from Central, North and South/Southeast Anatolia, Turkey. Zoonoses Public

Health. 2012 Mar;59(2):148-54.

[43] Jaijakul S, Arias CA, Hossain M, et al. Toscana meningoencephalitis: a comparison to other viral central nervous system infections. J Clin Virol. 2012 Nov;55(3):204-8.

[44] Percivalle E, Cassaniti I, Calzolari M, et al. Thirteen Years of Phleboviruses Circulation in Lombardy, a Northern Italy Region. Viruses. 2021 Jan 29;13(2).

[45] Mascitti H, Calin R, Dinh A, et al. Testicular pain associated with clear fluid meningitis: How many cases of Toscana virus are we missing? Int J Infect Dis. 2020 Apr;93:198-200.

[46] Zanelli G, Bianco C, Cusi MG. Testicular involvement during Toscana virus infection: an unusual manifestation? Infection. 2013 Jun;41(3):735-6.

[47] Hacioglu S, Dincer E, Isler CT, et al. A Snapshot Avian Surveillance Reveals West Nile Virus and Evidence of Wild Birds Participating in Toscana Virus Circulation. Vector Borne Zoonotic Dis. 2017 Oct;17(10):698-708.

[48] Gharsallah H, Tritar A, Naija H, et al. Severe meningoencephalomyelitis due to toscana virus: A diagnostic challenge. Travel Med Infect Dis. 2021 Sep-Oct;43:102131.

[49] Papa A, Paraforou T, Papakonstantinou I, et al. Severe encephalitis caused by Toscana virus, Greece. Emerg Infect Dis. 2014 Aug;20(8):1417-9.

[50] Rauch J, Zammarchi L, Corti G, et al. Serum cytokine and chemokine changes during Toscana virus meningitis. Med Microbiol Immunol. 2019 Dec;208(6):727-730.

[51] Navarro-Marí JM, Palop-Borrás B, Pérez-Ruiz M, et al. Serosurvey study of Toscana virus in domestic animals, Granada, Spain. Vector Borne Zoonotic Dis. 2011 May;11(5):583-7.

[52] Schwarz TF, Jäger G, Gilch S, et al. Serosurvey and laboratory diagnosis of imported sandfly fever virus, serotype Toscana, infection in Germany. Epidemiol Infect. 1995 Jun;114(3):501-10.

[53] Santos L, Cardoso MJ, Marinho AS, et al. [Seroprevalence survey of Toscana virus infection in Oporto region]. Acta Med Port. 2011 Dec;24 Suppl 2:479-82.

[54] Remoli ME, Fiorentini C, Marchi A, et al. Seroprevalence survey of arboviruses in workers from Tuscany, Italy. Med Lav. 2018 Jan 30;109(2):125-131.

[55] Saadawi WK, Abozaid FD, Almukhtar M, et al. Seroprevalence study of Toscana virus in Yafran area, Libya. J Vector Borne Dis. 2022 Apr-Jun;59(2):186-189.

[56] Marchi S, Trombetta CM, Kistner O, et al. Seroprevalence study of Toscana virus and viruses belonging to the Sandfly fever Naples antigenic complex in central and southern Italy. J Infect

Public Health. 2017 Nov-Dec;10(6):866-869.

[57] Pugliese A, Beltramo T, Torre D. Seroprevalence study of Tick-borne encephalitis, Borrelia burgdorferi, Dengue and Toscana virus in Turin Province. Cell Biochem Funct. 2007 Mar-Apr;25(2):185-8.

[58] Fezaa O, Bahri O, Alaya Bouafif NB, et al. Seroprevalence of Toscana virus infection in Tunisia. Int J Infect Dis. 2013 Dec;17(12):e1172-5.

[59] Tahir D, Alwassouf S, Loudahi A, et al. Seroprevalence of Toscana virus in dogs from Kabylia (Algeria). Clin Microbiol Infect. 2016 Mar;22(3):e16-7.

[60] Dahmani M, Alwassouf S, Grech-Angelini S, et al. Seroprevalence of Toscana virus in dogs from Corsica, France. Parasit Vectors. 2016 Jul 1;9(1):381.

[61] Brisbarre N, Attoui H, Gallian P, et al. Seroprevalence of Toscana virus in blood donors, France, 2007. Emerg Infect Dis. 2011 May;17(5):941-3.

[62] Ayhan N, López-Roig M, Monastiri A, et al. Seroprevalence of Toscana Virus and Sandfly Fever Sicilian Virus in European Bat Colonies Measured Using a Neutralization Test. Viruses. 2021 Jan 11;13(1).

[63] Anagnostou V, Papa A. Seroprevalence of Toscana virus among residents of Aegean Sea islands, Greece. Travel Med Infect Dis. 2013 Mar-Apr;11(2):98-102.

[64] Alwassouf S, Christodoulou V, Bichaud L, et al. Seroprevalence of Sandfly-Borne Phleboviruses Belonging to Three Serocomplexes (Sandfly fever Naples, Sandfly fever Sicilian and Salehabad) in Dogs from Greece and Cyprus Using Neutralization Test. PLoS Negl Trop Dis. 2016 Oct;10(10):e0005063.

[65] Shiraly R, Khosravi A, Farahangiz S. Seroprevalence of sandfly fever virus infection in military personnel on the western border of Iran. J Infect Public Health. 2017 Jan-Feb;10(1):59-63.

[66] Calamusa G, Valenti RM, Vitale F, et al. Seroprevalence of and risk factors for Toscana and Sicilian virus infection in a sample population of Sicily (Italy). J Infect. 2012 Feb;64(2):212-7.

[67] Martinez JG, García SG, Walter S, et al. Seroprevalence against Toscana virus in Spain: The case of the autonomous community of Madrid. J Vector Borne Dis. 2022 Apr-Jun;59(2):172-177.

[68] Valassina M, Valentini M, Pugliese A, et al. Serological survey of Toscana virus infections in a high-risk population in Italy. Clin Diagn Lab Immunol. 2003 May;10(3):483-4.

[69] Tezcan S, Dinçer E, Ülger M, et al. [Serological investigation of phlebovirus exposure in blood donors from the Mediterranean Province of Mersin, Turkey]. Mikrobiyol Bul. 2015 Jul;49(3):403-13.

[70] Amaro F, Luz T, Parreira P, et al. Serological evidence of Toscana virus infection in Portuguese patients. Epidemiol Infect. 2012 Jun;140(6):1147-50.

[71] Lelli D, Scanferla V, Moreno A, et al. Serological Evidence of Phleboviruses in Domestic Animals on the Pre-Apennine Hills (Northern Italy). Viruses. 2021 Aug 10;13(8).

[72] Maia C, Alwassouf S, Cristóvão JM, et al. Serological association between Leishmania infantum and sand fly fever Sicilian (but not Toscana) virus in sheltered dogs from southern Portugal. Parasit Vectors. 2017 Mar 13;10(1):92.

[73] Fezaa O, M'Ghirbi Y, Savellini GG, et al. Serological and molecular detection of Toscana and other Phleboviruses in patients and sandflies in Tunisia. BMC Infect Dis. 2014 Nov 15;14:598.

[74] Christova I, Panayotova E, Trifonova I, et al. Serologic evidence of widespread Toscana virus infection in Bulgaria. J Infect Public Health. 2020 Feb;13(2):164-166.

[75] Andayi F, Charrel RN, Kieffer A, et al. A sero-epidemiological study of arboviral fevers in Djibouti, Horn of Africa. PLoS Negl Trop Dis. 2014 Dec;8(12):e3299.

[76] Gonen OM, Sacagiu T. Sensory polymyeloradiculopathy associated with Toscana virus infection. J Neurovirol. 2013 Oct;19(5):508-10.

[77] Makranz C, Qutteineh H, Bin H, et al. Sandfly virus seroconversion associated with neurologic presentation. Neurol Neuroimmunol Neuroinflamm. 2016 Feb;3(1):e184.

[78] Nissen NB, Jespersen S, Vinner L, et al. [Sandfly virus meningitis in a Danish traveller returning from Tuscany]. Ugeskr Laeger. 2011 Oct 3;173(40):2505-6.

[79] Guler S, Guler E, Caglayik DY, et al. A sandfly fever virus outbreak in the East Mediterranean region of Turkey. Int J Infect Dis. 2012 Apr;16(4):e244-6.

[80] Papa A, Konstantinou G, Pavlidou V, et al. Sandfly fever virus outbreak in Cyprus. Clin Microbiol Infect. 2006 Feb;12(2):192-4.

[81] Ergünay K, Saygan MB, Aydoğan S, et al. Sandfly fever virus activity in central/northern Anatolia, Turkey: first report of Toscana virus infections. Clin Microbiol Infect. 2011 Apr;17(4):575-81.

[82] Imirzalioglu C, Schaller M, Bretzel RG. [Sandfly fever Naples virus (serotype Toscana)

infection with meningeal involvement after a vacation in Italy]. Dtsch Med Wochenschr. 2006 Dec 15;131(50):2838-40.

[83] Hemmersbach-Miller M, Parola P, Charrel RN, et al. Sandfly fever due to Toscana virus: an emerging infection in southern France. Eur J Intern Med. 2004 Aug;15(5):316-317.

[84] Eitrem R, Niklasson B, Weiland O. Sandfly fever among Swedish tourists. Scand J Infect Dis. 1991;23(4):451-7.

[85] Hukić M, Salimović-Besić I. Sandfly - Pappataci fever in Bosnia and Herzegovina: the new-old disease. Bosn J Basic Med Sci. 2009 Feb;9(1):39-43.

[86] Calzolari M, Romeo G, Munari M, et al. Sand Flies and Pathogens in the Lowlands of Emilia-Romagna (Northern Italy). Viruses. 2022 Oct 7;14(10).

[87] Bahri O, Fazaa O, Ben Alaya-Bouafif N, et al. [Role of Toscana virus in meningo-encephalitis in Tunisia]. Pathol Biol (Paris). 2011 Dec;59(6):e125-7.

[88] Dachraoui K, Chelbi I, Labidi I, et al. The Role of the Leishmania infantum Infected Dogs as a Potential Reservoir Host for Toscana Virus in a Zoonotic Visceral Leishmaniasis Focus of Northern Tunisia. Viruses. 2023 Apr 20;15(4).

[89] Sghaier W, Bahri O, Kedous E, et al. [Retrospective study of viral causes of central nervous system infections in Tunisia (2003-2009)]. Med Sante Trop. 2012 Oct-Dec;22(4):373-8.

[90] Valassina M, Cusi MG, Valensin PE. Rapid identification of Toscana virus by nested PCR during an outbreak in the Siena area of Italy. J Clin Microbiol. 1996 Oct;34(10):2500-2.

[91] De Lamballerie X, Tolou H, Durand JP, et al. Prevalence of Toscana virus antibodies in volunteer blood donors and patients with central nervous system infections in southeastern France. Vector Borne Zoonotic Dis. 2007 Summer;7(2):275-7.

[92] Papa A, Andriotis V, Tzilianos M. Prevalence of Toscana virus antibodies in residents of two Ionian islands, Greece. Travel Med Infect Dis. 2010 Sep;8(5):302-4.

[93] Punda-Polić V, Jerončić A, Mohar B, et al. Prevalence of Toscana virus antibodies in residents of Croatia. Clin Microbiol Infect. 2012 Jun;18(6):E200-3.

[94] Colomba C, Saporito L, Ciufolini MG, et al. Prevalence of Toscana sandfly fever virus antibodies in neurological patients and control subjects in Sicily. New Microbiol. 2012 Apr;35(2):161-5.

[95] Amodio E, Valentini M, Gori-Savellini G, et al. Prevalence of toscana and sicilian phlebovirus

antibodies in classic Kaposi sarcoma case patients and control subjects in sicily. J Infect Dis. 2011 Nov;204(9):1423-6.

[96] Anagnostou V, Papa A. Prevalence of antibodies to phleboviruses within the sand fly fever Naples virus species in humans, northern Greece. Clin Microbiol Infect. 2013 Jun;19(6):566-70.

[97] Leyes M, Ruiz de Gopegui E, Ribas M, et al. [Presence of the Toscana virus in Majorca. Prevalence and epidemiological characteristics in a hospital population]. Enferm Infecc Microbiol Clin. 2011 Apr;29(4):315-6.

[98] Sellali S, Lafri I, Hachid A, et al. Presence of the sandfly-borne phlebovirus (Toscana virus) in different bio-geographical regions of Algeria demonstrated by a microneutralisation-based seroprevalence study in owned dogs. Comp Immunol Microbiol Infect Dis. 2022 Sep;88:101861.

[99] Sakhria S, Alwassouf S, Fares W, et al. Presence of sandfly-borne phleboviruses of two antigenic complexes (Sandfly fever Naples virus and Sandfly fever Sicilian virus) in two different bio-geographical regions of Tunisia demonstrated by a microneutralisation-based seroprevalence study in dogs. Parasit Vectors. 2014 Oct 12;7:476.

[100] Dincer E, Gargari S, Ozkul A, et al. Potential animal reservoirs of Toscana virus and coinfections with Leishmania infantum in Turkey. Am J Trop Med Hyg. 2015 Apr;92(4):690-7.

[101] Remoli ME, Jiménez M, Fortuna C, et al. Phleboviruses detection in Phlebotomus perniciosus from a human leishmaniasis focus in South-West Madrid region, Spain. Parasit Vectors. 2016 Apr 13;9:205.

[102] Dachraoui K, Fares W, Bichaud L, et al. Phleboviruses associated with sand flies in arid bio-geographical areas of Central Tunisia. Acta Trop. 2016 Jun;158:13-19.

[103] Anagnostou V, Sdouga M, Volakli H, et al. Phlebovirus meningoencephalis complicated by Pseudomonas aeruginosa pneumonia: a case report. Vector Borne Zoonotic Dis. 2011 May;11(5):595-6.

[104] Papa A, Kontana A, Tsergouli K. Phlebovirus infections in Greece. J Med Virol. 2015 Jul;87(7):1072-6.

[105] Papa A, Kesisidou C, Kontana A, et al. Phlebovirus infection in Greece: a case report. Hippokratia. 2015 Apr-Jun;19(2):189-91.

[106] Ergunay K, Kasap OE, Orsten S, et al. Phlebovirus and Leishmania detection in sandflies from

eastern Thrace and northern Cyprus. Parasit Vectors. 2014 Dec 12;7:575.

[107] Braito A, Ciufolini MG, Pippi L, et al. Phlebotomus-transmitted toscana virus infections of the central nervous system: a seven-year experience in Tuscany. Scand J Infect Dis. 1998;30(5):505-8.

[108] Es-Sette N, Ajaoud M, Bichaud L, et al. Phlebotomus sergenti a common vector of Leishmania tropica and Toscana virus in Morocco. J Vector Borne Dis. 2014 Jun;51(2):86-90.

[109] Serata D, Rapinesi C, Del Casale A, et al. Personality changes after Toscana virus (TOSV) encephalitis in a 49-year-old man: A case report. Int J Neurosci. 2011 Mar;121(3):165-9.

[110] Ergünay K, Litzba N, Lo MM, et al. Performance of various commercial assays for the detection of Toscana virus antibodies. Vector Borne Zoonotic Dis. 2011 Jun;11(6):781-7.

[111] Ocal M, Orsten S, Inkaya AC, et al. Ongoing activity of Toscana virus genotype A and West Nile virus lineage 1 strains in Turkey: a clinical and field survey. Zoonoses Public Health. 2014 Nov;61(7):480-91.

[112] D'Ovidio MC, Venturi G, Fiorentini C, et al. Occupational risk associated with Toscana virus infection in Tuscany, Italy. Occup Med (Lond). 2008 Dec;58(8):540-4.

[113] Valentini M, Valassina M, Savellini GG, et al. Nucleotide variability of Toscana virus M segment in strains isolated from clinical cases. Virus Res. 2008 Jul;135(1):187-90.

[114] Greco F, Mauro MV, Tenuta R, et al. A new case of meningitis due to Toscana virus. New Microbiol. 2012 Jan;35(1):99-100.

[115] Alwassouf S, Maia C, Ayhan N, et al. Neutralization-based seroprevalence of Toscana virus and sandfly fever Sicilian virus in dogs and cats from Portugal. J Gen Virol. 2016 Nov;97(11):2816-2823.

[116] Ehrnst A, Peters CJ, Niklasson B, et al. Neurovirulent Toscana virus (a sandfly fever virus) in Swedish man after visit to Portugal. Lancet. 1985 May 25;1(8439):1212-3.

[117] Papa A, Mallias J, Tsergouli K, et al. Neuroinvasive phlebovirus infection in Greece: a case report. Intervirology. 2014;57(6):393-5.

[118] Schwarz TF, Jäger G, Gilch S, et al. Nested RT-PCR for detection of sandfly fever virus, serotype Toscana, in clinical specimens, with confirmation by nucleotide sequence analysis. Res Virol. 1995 Sep-Oct;146(5):355-62.

[119] Es-Sette N, Ajaoud M, Charrel RN, et al. [Molecular epidemiology of phlebovirus in four

provinces in Morocco]. Bull Soc Pathol Exot. 2016 Aug;109(3):143-50.

[120] Epelboin L, Hausfater P, Schuffenecker I, et al. Meningoencephalitis due to Toscana virus in a French traveler returning from central Italy. J Travel Med. 2008 Sep-Oct;15(5):361-3.

[121] Varani S, Gelsomino F, Bartoletti M, et al. Meningitis Caused by Toscana Virus Is Associated with Strong Antiviral Response in the CNS and Altered Frequency of Blood Antigen-Presenting Cells. Viruses. 2015 Nov 11;7(11):5831-43.

[122] Beersma MF, Grimbergen YA, Kroon FP, et al. [Meningitis caused by Toscana virus during a summer stay in Italy]. Ned Tijdschr Geneeskd. 2004 Feb 7;148(6):286-8.

[123] Schwarz TF, Pfister HW, Schwarz J, et al. Meningitis caused by sandfly fever virus after returning from the Mediterranean region. Nervenarzt. 1995;66(10):789-791.

[124] Navarro JM, Fernández-Roldán C, Pérez-Ruiz M, et al. [Meningitis by Toscana virus in Spain: description of 17 cases]. Med Clin (Barc). 2004 Mar 27;122(11):420-2.

[125] Tschumi F, Schmutz S, Kufner V, et al. Meningitis and epididymitis caused by Toscana virus infection imported to Switzerland diagnosed by metagenomic sequencing: a case report. BMC Infect Dis. 2019 Jul 8;19(1):591.

[126] Pérez-Ruiz M, Navarro-Marí JM, Sánchez-Seco MP, et al. Lymphocytic choriomeningitis virus-associated meningitis, southern Spain. Emerg Infect Dis. 2012 May;18(5):855-8.

[127] Bartels S, de Boni L, Kretzschmar HA, et al. Lethal encephalitis caused by the Toscana virus in an elderly patient. J Neurol. 2012 Jan;259(1):175-7.

[128] Soldateschi D, dal Maso GM, Valassina M, et al. Laboratory diagnosis of Toscana virus infection by enzyme immunoassay with recombinant viral nucleoprotein. J Clin Microbiol. 1999 Mar;37(3):649-52.

[129] Nougairede A, Bichaud L, Thiberville SD, et al. Isolation of Toscana virus from the cerebrospinal fluid of a man with meningitis in Marseille, France, 2010. Vector Borne Zoonotic Dis. 2013 Sep;13(9):685-8.

[130] Calzolari M, Chiapponi C, Bellini R, et al. Isolation of three novel reassortant phleboviruses, Ponticelli I, II, III, and of Toscana virus from field-collected sand flies in Italy. Parasit Vectors. 2018 Feb 6;11(1):84.

[131] Özbel Y, Oğuz G, Arserim SK, et al. The initial detection of Toscana virus in phlebotomine sandflies from Turkey. Med Vet Entomol. 2020 Dec;34(4):402-410.

[132] Matusali G, D'Abramo A, Terrosi C, et al. Infectious Toscana Virus in Seminal Fluid of Young Man Returning from Elba Island, Italy. Emerg Infect Dis. 2022 Apr;28(4):865-869.

[133] Mendoza-Montero J, Gámez-Rueda MI, Navarro-Marí JM, et al. Infections due to sandfly fever virus serotype Toscana in Spain. Clin Infect Dis. 1998 Sep;27(3):434-6.

[134] Sonderegger B, Hachler H, Dobler G, et al. Imported aseptic meningitis due to Toscana virus acquired on the island of Elba, Italy, August 2008. Euro Surveill. 2009 Jan 8;14(1).

[135] Amodio E, Cusi MG, Valenti RM, et al. Immunoglobulin M seropositivity for Toscana virus in a random population sample in Sicily. Int J Infect Dis. 2012 Aug;16(8):e633-5.

[136] Daoudi M, Calzolari M, Boussaa S, et al. Identification of Toscana virus in natural population of sand flies (Diptera: Psychodidae) from Moroccan leishmaniasis foci. J Infect Public Health. 2022 Apr;15(4):406-411.

[137] Maia C, Ayhan N, Cristóvão JM, et al. Human seroprevalence of Toscana virus and Sicilian phlebovirus in the southwest of Portugal. Eur J Clin Microbiol Infect Dis. 2022 Jan;41(1):137-141.

[138] Calzolari M, Angelini P, Finarelli AC, et al. Human and entomological surveillance of Toscana virus in the Emilia-Romagna region, Italy, 2010 to 2012. Euro Surveill. 2014 Dec 4;19(48):20978.

[139] Ranaldi R, Goteri G, Biagetti S, et al. Histological description of the lymphadenopathy related to Toscana virus infection. Report of a case. Pathol Res Pract. 2011 Mar 15;207(3):197-201.

[140] Ayhan N, Sherifi K, Taraku A, et al. High Rates of Neutralizing Antibodies to Toscana and Sandfly Fever Sicilian Viruses in Livestock, Kosovo. Emerg Infect Dis. 2017 Jun;23(6):989-992.

[141] Ayhan N, Rodríguez-Teijeiro JD, López-Roig M, et al. High rates of antibodies against Toscana and Sicilian phleboviruses in common quail Coturnix coturnix birds. Front Microbiol. 2022;13:1091908.

[142] Eitrem R, Stylianou M, Niklasson B. High prevalence rates of antibody to three sandfly fever viruses (Sicilian, Naples, and Toscana) among Cypriots. Epidemiol Infect. 1991 Dec;107(3):685-91.

[143] Rota E, Morelli N, Immovilli P, et al. Guillain-Barré-like axonal polyneuropathy associated with Toscana virus infection: A case report. Medicine (Baltimore). 2017 Sep;96(38):e8081.

[144] Collao X, Palacios G, de Ory F, et al. Granada virus: a natural phlebovirus reassortant of the sandfly fever Naples serocomplex with low seroprevalence in humans. Am J Trop Med Hyg. 2010 Oct;83(4):760-5.

[145] Collao X, Palacios G, Sanbonmatsu-Gámez S, et al. Genetic diversity of Toscana virus. Emerg Infect Dis. 2009 Apr;15(4):574-7.

[146] Schultze D, Korte W, Rafeiner P, et al. First report of sandfly fever virus infection imported from Malta into Switzerland, October 2011. Euro Surveill. 2012 Jul 5;17(27).

[147] Es-Sette N, Nourlil J, Hamdi S, et al. First detection of Toscana virus RNA from sand flies in the genus Phlebotomus (Diptera: Phlebotomidae) naturally infected in Morocco. J Med Entomol. 2012 Nov;49(6):1507-9.

[148] Bichaud L, Izri A, de Lamballerie X, et al. First detection of Toscana virus in Corsica, France. Clin Microbiol Infect. 2014 Feb;20(2):O101-4.

[149] Valassina M, Valentini M, Valensin PE, et al. Fast duplex one-step RT-PCR for rapid differential diagnosis of entero- or toscana virus meningitis. Diagn Microbiol Infect Dis. 2002 Jul;43(3):201-5.

[150] Schirmer L, Wölfel S, Georgi E, et al. Extensive Recruitment of Plasma Blasts to the Cerebrospinal Fluid in Toscana Virus Encephalitis. Open Forum Infect Dis. 2015 Sep;2(3):ofv124.

[151] Ortuño M, Muñoz C, Spitzová T, et al. Exposure to Phlebotomus perniciosus sandfly vectors is positively associated with Toscana virus and Leishmania infantum infection in human blood donors in Murcia Region, southeast Spain. Transbound Emerg Dis. 2022 Sep;69(5):e1854-e1864.

[152] Valassina M, Cuppone AM, Bianchi S, et al. Evidence of Toscana virus variants circulating in Tuscany, Italy, during the summers of 1995 to 1997. J Clin Microbiol. 1998 Jul;36(7):2103-4.

[153] Braito A, Corbisiero R, Corradini S, et al. Evidence of Toscana virus infections without central nervous system involvement: a serological study. Eur J Epidemiol. 1997 Oct;13(7):761-4.

[154] Francisci D, Papili R, Camanni G, et al. Evidence of Toscana virus circulation in Umbria: first report. Eur J Epidemiol. 2003;18(5):457-9.

[155] Punda-Polić V, Mohar B, Duh D, et al. Evidence of an autochthonous Toscana virus strain in Croatia. J Clin Virol. 2012 Sep;55(1):4-7.

[156] Benbetka C, Hachid A, Benallal KE, et al. Epidemiology, Isolation, and Genetic Characterization of Toscana Virus in Algerian Patients Displaying Neurological Infection, 2016-2018. IJID Reg. 2023 Jun;7:193-198.

[157] Gori Savellini G, Gandolfo C, Cusi MG. Epidemiology of Toscana virus in South Tuscany over the years 2011-2019. J Clin Virol. 2020 Jul;128:104452.

[158] Bichaud L, Souris M, Mary C, et al. Epidemiologic relationship between Toscana virus infection and Leishmania infantum due to common exposure to Phlebotomus perniciosus sandfly vector. PLoS Negl Trop Dis. 2011 Sep;5(9):e1328.

[159] Dionisio D, Valassina M, Ciufolini MG, et al. Encephalitis without meningitis due to sandfly fever virus serotype toscana. Clin Infect Dis. 2001 Apr 15;32(8):1241-3.

[160] Popescu CP, Cotar AI, Dinu S, et al. Emergence of Toscana Virus, Romania, 2017-2018. Emerg Infect Dis. 2021 May;27(5):1482-1485.

[161] Erdem H, Ergunay K, Yilmaz A, et al. Emergence and co-infections of West Nile virus and Toscana virus in Eastern Thrace, Turkey. Clin Microbiol Infect. 2014 Apr;20(4):319-25.

[162] Verani P, Ciufolini MG, Caciolli S, et al. Ecology of viruses isolated from sand flies in Italy and characterized of a new Phlebovirus (Arabia virus). Am J Trop Med Hyg. 1988 Mar;38(2):433-9.

[163] Verani P, Ciufolini MG, Nicoletti L, et al. [Ecological and epidemiological studies of Toscana virus, an arbovirus isolated from Phlebotomus]. Ann Ist Super Sanita. 1982;18(3):397-9.

[164] Peyrefitte CN, Grandadam M, Bessaud M, et al. Diversity of Phlebotomus perniciosus in Provence, southeastern France: Detection of two putative new phlebovirus sequences. Vector Borne Zoonotic Dis. 2013 Sep;13(9):630-6.

[165] Ciufolini MG, Fiorentini C, di Bonito P, et al. Detection of Toscana virus-specific immunoglobulins G and M by an enzyme-linked immunosorbent assay based on recombinant viral nucleoprotein. J Clin Microbiol. 1999 Jun;37(6):2010-2.

[166] Arden KE, Heney C, Shaban B, et al. Detection of Toscana virus from an adult traveler returning to Australia with encephalitis. J Med Virol. 2017 Oct;89(10):1861-1864.

[167] Venturi G, Madeddu G, Rezza G, et al. Detection of Toscana virus central nervous system infections in Sardinia Island, Italy. J Clin Virol. 2007 Sep;40(1):90-1.

[168] Morini S, Calzolari M, Rossini G, et al. Detection of Specific Antibodies against Toscana Virus

among Blood Donors in Northeastern Italy and Correlation with Sand Fly Abundance in 2014. Microorganisms. 2020 Jan 21;8(2).

[169] Valassina M, Meacci F, Valensin PE, et al. Detection of neurotropic viruses circulating in Tuscany: the incisive role of Toscana virus. J Med Virol. 2000 Jan;60(1):86-90.

[170] Martínez-García FA, Moreno-Docón A, Segovia-Hernández M, et al. [Deafness as a sequela of Toscana virus meningitis]. Med Clin (Barc). 2008 May 3;130(16):639.

[171] Houghton R, Gori Savellini G, Chen H, et al. Comparison of a new prototype immunochromatographic assay and a commercial enzyme-linked immunosorbent assay for the detection of serum antibodies against Toscana virus. J Virol Methods. 2013 Jan;187(1):182-4.

[172] Ayhan N, Alten B, Ivovic V, et al. Cocirculation of Two Lineages of Toscana Virus in Croatia. Front Public Health. 2017;5:336.

[173] Charrel RN, Izri A, Temmam S, et al. Cocirculation of 2 genotypes of Toscana virus, southeastern France. Emerg Infect Dis. 2007 Mar;13(3):465-8.

[174] Sakhria S, Bichaud L, Mensi M, et al. Co-circulation of Toscana virus and Punique virus in northern Tunisia: a microneutralisation-based seroprevalence study. PLoS Negl Trop Dis. 2013;7(9):e2429.

[175] Fares W, Dachraoui K, Barhoumi W, et al. Co-circulation of Toscana virus and Leishmania infantum in a focus of zoonotic visceral leishmaniasis from Central Tunisia. Acta Trop. 2020 Apr;204:105342.

[176] Calzolari M, Romeo G, Callegari E, et al. Co-Circulation of Phleboviruses and Leishmania Parasites in Sand Flies from a Single Site in Italy Monitored between 2017 and 2020. Viruses. 2021 Aug 21;13(8).

[177] Calzolari M, Ferrarini G, Bonilauri P, et al. Co-circulation of eight different phleboviruses in sand flies collected in the Northern Apennine Mountains (Italy). Infect Genet Evol. 2018 Oct;64:131-134.

[178] Vilibic-Cavlek T, Zidovec-Lepej S, Ledina D, et al. Clinical, Virological, and Immunological Findings in Patients with Toscana Neuroinvasive Disease in Croatia: Report of Three Cases. Trop Med Infect Dis. 2020 Sep 14;5(3).

[179] Petersen PT, Bodilsen J, Jepsen MPG, et al. Clinical features and prognostic factors in adults with viral meningitis. Brain. 2023 Sep 1;146(9):3816-3825.

[180] Navarro-Marí JM, Gómez-Camarasa C, Pérez-Ruiz M, et al. Clinic-epidemiologic study of human infection by Granada virus, a new phlebovirus within the sandfly fever Naples serocomplex. Am J Trop Med Hyg. 2013 May;88(5):1003-6.

[181] Masse S, Ayhan N, Capai L, et al. Circulation of Toscana Virus in a Sample Population of Corsica, France. Viruses. 2019 Sep 4;11(9).

[182] Pierro A, Ficarelli S, Ayhan N, et al. Characterization of antibody response in neuroinvasive infection caused by Toscana virus. Clin Microbiol Infect. 2017 Nov;23(11):868-873.

[183] Carhan A, Uyar Y, Ozkaya E, et al. Characterization of a sandfly fever Sicilian virus isolated during a sandfly fever epidemic in Turkey. J Clin Virol. 2010 Aug;48(4):264-9.

[184] Nicoletti L, Verani P, Caciolli S, et al. Central nervous system involvement during infection by Phlebovirus toscana of residents in natural foci in central Italy (1977-1988). Am J Trop Med Hyg. 1991 Oct;45(4):429-34.

[185] Martinez-Garcia FA, Moreno-Docon A, Lopez-Lopez M, et al. [A case of meningitis due to Toscana virus in Murcia]. Rev Neurol. 2007 Sep 1-15;45(5):317-8.

[186] Ergunay K, Ismayilova V, Colpak IA, et al. A case of central nervous system infection due to a novel Sandfly Fever Virus (SFV) variant: Sandfly Fever Turkey Virus (SFTV). J Clin Virol. 2012 May;54(1):79-82.

[187] Dincer E, Karapinar Z, Oktem M, et al. Canine Infections and Partial S Segment Sequence Analysis of Toscana Virus in Turkey. Vector Borne Zoonotic Dis. 2016 Sep;16(9):611-8.

[188] Schwarz TF, Gilch S, Jäger G. Aseptic meningitis caused by sandfly fever virus, serotype Toscana. Clin Infect Dis. 1995 Sep;21(3):669-71.

[189] Venturi G, El-Sawaf G, Arpino C, et al. Arboviral infections in Egyptian and Sardinian children and adults with aseptic meningitis and meningo-encephalitis. Scand J Infect Dis. 2009;41(11-12):898-9.

[190] Pereira A, Ayhan N, Cristóvão JM, et al. Antibody Response to Toscana Virus and Sandfly Fever Sicilian Virus in Cats Naturally Exposed to Phlebotomine Sand Fly Bites in Portugal. Microorganisms. 2019 Sep 11;7(9).

[191] de Ory-Manchón F, Sanz-Moreno JC, Aranguez-Ruiz E, et al. [Age-dependent seroprevalence of Toscana virus in the Community of Madrid: 1993-1994 and 1999-2000]. Enferm Infecc Microbiol Clin. 2007 Mar;25(3):187-9.

[192] Terrosi C, Olivieri R, Bianco C, et al. Age-dependent seroprevalence of Toscana virus in central Italy and correlation with the clinical profile. Clin Vaccine Immunol. 2009 Aug;16(8):1251-2.

[193] Marlinge M, Crespy L, Zandotti C, et al. Afebrile meningoencephalitis with transient central facial paralysis due to Toscana virus infection, southeastern France, 2014 [corrected]. Euro Surveill. 2014 Dec 4;19(48):20974.

[194] Tappe D, Schmidt-Chanasit J, Günther S, et al. Acute Toscana virus infection mimicked by Yersinia-induced reactive arthritis syndrome after journey to Spain. J Clin Virol. 2010 Jan;47(1):104-5.

[195] Kuşcu F, Menemenlioğlu D, Oztürk DB, et al. [Acute Toscana virus infection in an anti-HIV positive patient]. Mikrobiyol Bul. 2014 Jan;48(1):168-73.

[196] Defuentes G, Rapp C, Imbert P, et al. Acute meningitis owing to phlebotomus fever Toscana virus imported to France. J Travel Med. 2005 Sep-Oct;12(5):295-6.

[197] Echevarría JM, de Ory F, Guisasola ME, et al. Acute meningitis due to Toscana virus infection among patients from both the Spanish Mediterranean region and the region of Madrid. J Clin Virol. 2003 Jan;26(1):79-84.

[198] Charrel RN, Petzold GC, Oechtering J. Acute hydrocephalus due to impaired CSF resorption in Toscana virus meningoencephalitis. Neurology. 2013 Apr 9;80(15):1444.

[199] Suardi LR, Di Lauria N, Pozzi M, et al. Acute cerebellar ataxia: a rare Toscana Virus (TOSV) meningoencephalitis complication. Int J Neurosci. 2020 Mar;130(3):276-278.

[200] Abbas MAS, Lachheb J, Chelbi I, et al. Independent Circulation of Leishmania major and Leishmania tropica in Their Respective Sandfly Vectors for Transmission of Zoonotic and Chronic Cutaneous Leishmaniasis Co-Existing in a Mixed Focus of Central Tunisia. Pathogens. 2022 Jul 29;11(8).

[201] Abbate JM, Maia C, Pereira A, et al. Identification of trypanosomatids and blood feeding preferences of phlebotomine sand fly species common in Sicily, Southern Italy. PLoS One. 2020;15(3):e0229536.

[202] Absavaran A, Rassi Y, Parvizi P, et al. Identification of Sand flies of the Subgenus Larroussius based on Molecular and Morphological Characters in North Western Iran. Iran J Arthropod Borne Dis. 2009;3(2):22-35.

[203] Afonso MO, Campino L, Cortes S, et al. The phlebotomine sandflies of Portugal. XIII--

Occurrence of Phlebotomus sergenti Parrot, 1917 in the Arrabida leishmaniasis focus. Parasite. 2005 Mar;12(1):69-72.

[204] Ajaoud M, Es-Sette N, Charrel RN, et al. Phlebotomus sergenti in a cutaneous leishmaniasis focus in Azilal province (High Atlas, Morocco): molecular detection and genotyping of Leishmania tropica, and feeding behavior. PLoS Negl Trop Dis. 2015 Mar;9(3):e0003687.

[205] Akhoundi M, Bakhtiari R, Guillard T, et al. Diversity of the bacterial and fungal microflora from the midgut and cuticle of phlebotomine sand flies collected in North-Western Iran. PLoS One. 2012;7(11):e50259.

[206] Akhoundi M, Mirzaei A, Baghaei A, et al. Sand fly (Diptera: Psychodidae) distribution in the endemic and non-endemic foci of visceral leishmaniasis in northwestern Iran. J Vector Ecol. 2013 Jun;38(1):97-104.

[207] Alcover MM, Ballart C, Martín-Sánchez J, et al. Factors influencing the presence of sand flies in Majorca (Balearic Islands, Spain) with special reference to Phlebotomus pernicious, vector of Leishmania infantum. Parasit Vectors. 2014 Sep 4;7:421.

[208] Alcover MM, Ballart C, Serra T, et al. Temporal trends in canine leishmaniosis in the Balearic Islands (Spain): a veterinary questionnaire. Prospective canine leishmaniosis survey and entomological studies conducted on the Island of Minorca, 20 years after first data were obtained. Acta Trop. 2013 Dec;128(3):642-51.

[209] Alkan C, Alwassouf S, Piorkowski G, et al. Isolation, genetic characterization, and seroprevalence of Adana virus, a novel phlebovirus belonging to the Salehabad virus complex, in Turkey. J Virol. 2015 Apr;89(8):4080-91.

[210] Alten B, Maia C, Afonso MO, et al. Seasonal Dynamics of Phlebotomine Sand Fly Species Proven Vectors of Mediterranean Leishmaniasis Caused by Leishmania infantum. PLoS Negl Trop Dis. 2016 Feb;10(2):e0004458.

[211] Amaro F, Zé-Zé L, Alves MJ, et al. Co-circulation of a novel phlebovirus and Massilia virus in sandflies, Portugal. Virol J. 2015 Oct 24;12:174.

[212] Amira A, Bounamous A, Kouba Y, et al. Sand Flies (Diptera: Psychodidae): Fauna and Ecology in the Northeast of Algeria. J Med Entomol. 2022 May 11;59(3):855-864.

[213] Arfuso F, Gaglio G, Abbate JM, et al. Identification of phlebotomine sand flies through MALDI-TOF mass spectrometry and in-house reference database. Acta Tropica. 2019

2019/06/01/;194:47-52.

[214] Ascoli V, Senis G, Zucchetto A, et al. Distribution of 'promoter' sandflies associated with incidence of classic Kaposi's sarcoma. Med Vet Entomol. 2009 Sep;23(3):217-25.

[215] Balaska S, Calzolari M, Grisendi A, et al. Monitoring of Insecticide Resistance Mutations and Pathogen Circulation in Sand Flies from Emilia-Romagna, a Leishmaniasis Endemic Region of Northern Italy. Viruses. 2023 Jan 3;15(1).

[216] Baldelli R, Piva S, Salvatore D, et al. Canine leishmaniasis surveillance in a northern Italy kennel. Vet Parasitol. 2011 Jun 30;179(1-3):57-61.

[217] Ballart C, Barón S, Alcover MM, et al. Distribution of phlebotomine sand flies (Diptera: Psychodidae) in Andorra: first finding of P. perniciosus and wide distribution of P. ariasi. Acta Trop. 2012 Apr;122(1):155-9.

[218] Ballart C, Guerrero I, Castells X, et al. Importance of individual analysis of environmental and climatic factors affecting the density of Leishmania vectors living in the same geographical area: the example of Phlebotomus ariasi and P. perniciosus in northeast Spain. Geospat Health. 2014 May;8(2):389-403.

[219] Ballart C, Pesson B, Gállego M. Isoenzymatic characterization of Phlebotomus ariasi and P. perniciosus of canine leishmaniasis foci from Eastern Pyrenean regions and comparison with other populations from Europe. Parasite. 2018;25:3.

[220] Barhoumi W, Chelbi I, Zhioua E. [Effects of the development of irrigation systems in the arid areas on the establishment of Phlebotomus (Larroussius) perfiliewi Parrot, 1939]. Bull Soc Pathol Exot. 2012 Dec;105(5):403-5.

[221] Barhoumi W, Fares W, Cherni S, et al. Changes of Sand Fly Populations and Leishmania infantum Infection Rates in an Irrigated Village Located in Arid Central Tunisia. Int J Environ Res Public Health. 2016 Mar 16;13(3).

[222] Barhoumi W, Qualls WA, Archer RS, et al. Irrigation in the arid regions of Tunisia impacts the abundance and apparent density of sand fly vectors of Leishmania infantum. Acta Trop. 2015 Jan;141(Pt A):73-8.

[223] Benabid M, Ghrab J, Rhim A, et al. Temporal dynamics and Leishmania infantum infection prevalence of Phlebotomus perniciosus (Diptera, Phlebotominae) in highly endemic areas of visceral leishmaniasis in Tunisia. PLoS One. 2017;12(9):e0184700.

[224] Benallal KE, Benikhlef R, Garni R, et al. Presence of Phlebotomus perniciosus Atypical Form in Algeria. J Arthropod Borne Dis. 2017 Mar;11(1):139-146.

[225] Benante JP, Fox J, Lawrence K, et al. A Comparative Study of Mosquito and Sand Fly (Diptera: Psychodidae: Phlebotominae) Sampling Using Dry Ice and Chemically Generated Carbon Dioxide From Three Different Prototype CO2 Generators. J Econ Entomol. 2019 Feb 12;112(1):494-498.

[226] Beniklef R, Aoun K, Boudrissa K, et al. Cutaneous Leishmaniasis in Algeria; Highlight on the Focus of M'Sila. Microorganisms. 2021 Apr 29;9(5).

[227] Bennai K, Tahir D, Lafri I, et al. Molecular detection of Leishmania infantum DNA and host blood meal identification in Phlebotomus in a hypoendemic focus of human leishmaniasis in northern Algeria. PLoS Negl Trop Dis. 2018 Jun;12(6):e0006513.

[228] Berchi S, Bounamous A, Louadi K, et al. Morphological distinction between two sympatric species: Phlebotomus pernlciosus and Phlebotomus longicuspis (Diptera: Psychodidae). Ann Soc Entomol France. 2007 04/01;43:201-203.

[229] Berdjane-Brouk Z, Charrel RN, Bitam I, et al. Record of Phlebotomus (Transphlebotomus) mascittii Grassi, 1908 and Phlebotomus (Larroussius) chadlii Rioux, Juminer & Gibily, 1966 female in Algeria. Parasite. 2011 Nov;18(4):337-9.

[230] Bosnić S, Gradoni L, Khoury C, et al. A review of leishmaniasis in Dalmatia (Croatia) and results from recent surveys on phlebotomine sandflies in three southern counties. Acta Trop. 2006 Aug;99(1):42-9.

[231] Bouattour A, Amri A, Belkhiria JA, et al. Canine leishmaniosis in Tunisia: Growing prevalence, larger zones of infection. PLoS Negl Trop Dis. 2021 Dec;15(12):e0009990.

[232] Boudrissa A, Cherif K, Kherrachi I, et al. [Spread of Leishmania major to the north of Algeria]. Bull Soc Pathol Exot. 2012 Feb;105(1):30-5.

[233] Boussaa S, Kahime K, Samy AM, et al. Species composition of sand flies and bionomics of Phlebotomus papatasi and P. sergenti (Diptera: Psychodidae) in cutaneous leishmaniasis endemic foci, Morocco. Parasit Vectors. 2016 Feb 2;9:60.

[234] Boussaa S, Neffa M, Pesson B, et al. Phlebotomine sandflies (Diptera: Psychodidae) of southern Morocco: results of entomological surveys along the Marrakech-Ouarzazat and Marrakech-Azilal roads. Ann Trop Med Parasitol. 2010 Mar;104(2):163-70.

[235] Boussaa S, Pesson B, Boumezzough A. Faunistic study of the sandflies (Diptera: Psychodidae) in an emerging focus of cutaneous leishmaniasis in Al Haouz province, Morocco. Ann Trop Med Parasitol. 2009 Jan;103(1):73-83.

[236] Branco S, Alves-Pires C, Maia C, et al. Entomological and ecological studies in a new potential zoonotic leishmaniasis focus in Torres Novas municipality, Central Region, Portugal. Acta Trop. 2013 Mar;125(3):339-48.

[237] Bravo-Barriga D, Parreira R, Maia C, et al. Detection of Leishmania DNA and blood meal sources in phlebotomine sand flies (Diptera: Psychodidae) in western of Spain: Update on distribution and risk factors associated. Acta Trop. 2016 Dec;164:414-424.

[238] Calzolari M, Carra E, Rugna G, et al. Isolation and Molecular Typing of Leishmania infantum from Phlebotomus perfiliewi in a Re-Emerging Focus of Leishmaniasis, Northeastern Italy. Microorganisms. 2019 Dec 3;7(12).

[239] Cazan CD, Păstrav IR, Györke A, et al. Seasonal dynamics of a population of Phlebotomus (Larroussius) perfiliewi Parrot, 1930 (Diptera: Psychodidae) in North-Eastern Romania. Parasitol Res. 2019 May;118(5):1371-1384.

[240] Cazan CD, Păstrav IR, Ionică AM, et al. Updates on the distribution and diversity of sand flies (Diptera: Psychodidae) in Romania. Parasit Vectors. 2019 May 20;12(1):247.

[241] Chargui N, Slama D, Haouas N, et al. Transmission cycle analysis in a Leishmania infantum focus: Infection rates and blood meal origins in sand flies (Diptera: Psychodidae). J Vector Ecol. 2018 Dec;43(2):321-327.

[242] Charrel RN, Moureau G, Temmam S, et al. Massilia virus, a novel Phlebovirus (Bunyaviridae) isolated from sandflies in the Mediterranean. Vector Borne Zoonotic Dis. 2009 Oct;9(5):519-30.

[243] Chaskopoulou A, Miaoulis M, Kashefi J. Ground ultra low volume (ULV) space spray applications for the control of wild sand fly populations (Psychodidae: Phlebotominae) in Europe. Acta Trop. 2018 Jun;182:54-59.

[244] Chelbi I, Abdi A, Depaquit J, et al. Investigation of the Sandfly Fauna of Central Arid Areas and Northern Humid Regions of Tunisia, with Morphological and Molecular Identification of the Recently Established Population of Phlebotomus (Larroussius) perfiliewi. Insects. 2022 Nov 16;13(11).

[245] Cotteaux-Lautard C, Leparc-Goffart I, Berenger JM, et al. Phenology and host preferences Phlebotomus perniciosus (Diptera: Phlebotominae) in a focus of Toscana virus (TOSV) in South of France. Acta Trop. 2016 Jan;153:64-9.

[246] Dachraoui K, Chelbi I, Ben Said M, et al. Transmission Dynamics of Punique Virus in Tunisia. Viruses. 2022 Apr 26;14(5).

[247] Dantas-Torres F, Tarallo VD, Latrofa MS, et al. Ecology of phlebotomine sand flies and Leishmania infantum infection in a rural area of southern Italy. Acta Trop. 2014 Sep;137:67-73.

[248] Darkaoui N, Janati Idrissi A, Talbi FZ, et al. Seasonal Dynamics of Sand Flies (Diptera, Pshycodidae), Vectors of Cutaneous Leishmaniasis, in the City of Fez, Northern Morocco. ScientificWorldJournal. 2022;2022:4095129.

[249] Dehkordi AS, Rassi Y, Oshaghi M, et al. Molecular Detection of Leishmania infantum in Naturally Infected Phlebotomus perfiliewi transcaucasicus in Bilesavar District, Northwestern Iran. Iran J Arthropod Borne Dis. 2011;5(1):20-7.

[250] Demir S, Karakuş M. Natural Leishmania infection of Phlebotomus sergenti (Diptera: Phlebotominae) in an endemic focus of cutaneous leishmaniasis in Şanlıurfa, Turkey. Acta Trop. 2015 Sep;149:45-8.

[251] Dereure J, Vanwambeke SO, Malé P, et al. The potential effects of global warming on changes in canine leishmaniasis in a focus outside the classical area of the disease in southern France. Vector Borne Zoonotic Dis. 2009 Dec;9(6):687-94.

[252] Díaz-Sáez V, Corpas-López V, Merino-Espinosa G, et al. Seasonal dynamics of phlebotomine sand flies and autochthonous transmission of Leishmania infantum in high-altitude ecosystems in southern Spain. Acta Trop. 2021 Jan;213:105749.

[253] Díaz-Sáez V, Morillas-Mancilla MJ, Corpas-López V, et al. Leishmaniasis vectors in the environment of treated leishmaniasis cases in Spain. Transbound Emerg Dis. 2022 Nov;69(6):3247-3255.

[254] Dokianakis E, Tsirigotakis N, Christodoulou V, et al. DNA sequencing confirms PCR-RFLP identification of wild caught Larroussius sand flies from Crete and Cyprus. Acta Trop. 2016 Dec;164:314-320.

[255] El-Mouhdi K, Fekhaoui M, Chahlaoui A, et al. Entomological Exploration of Sand Flies in Human Communities Affected by Cutaneous and Visceral Leishmaniasis in El Hajeb Province,

Morocco. ScientificWorldJournal. 2023;2023:4628625.

[256] Ergunay K, Erisoz Kasap O, Kocak Tufan Z, et al. Molecular evidence indicates that Phlebotomus major sensu lato (Diptera: Psychodidae) is the vector species of the recently-identified sandfly fever Sicilian virus variant: sandfly fever turkey virus. Vector Borne Zoonotic Dis. 2012 Aug;12(8):690-8.

[257] erisoz kasap O, Belen AS, Kaynas S, et al. Activity Patterns of Sand Fly (Diptera: Psychodidae) Species and Comparative Performance of Different Traps in an Endemic Cutaneous Leishmaniasis Focus in Cukurova Plain, Southern Anatolia, Turkey. Acta Veterinaria Brno. 2009 06/01;78.

[258] Fares W, Charrel RN, Dachraoui K, et al. Infection of sand flies collected from different bio-geographical areas of Tunisia with phleboviruses. Acta Trop. 2015 Jan;141(Pt A):1-6.

[259] Farkas R, Tánczos B, Bongiorno G, et al. First surveys to investigate the presence of canine leishmaniasis and its phlebotomine vectors in Hungary. Vector Borne Zoonotic Dis. 2011 Jul;11(7):823-34.

[260] Faucher B, Bichaud L, Charrel R, et al. Presence of sandflies infected with Leishmania infantum and Massilia virus in the Marseille urban area. Clin Microbiol Infect. 2014 May;20(5):O340-3.

[261] Ferroglio E, Romano A, Dettoni F, et al. Distribution of Phlebotomus perniciosus in North-Italy: a study on 18S rDNA of phlebotomine sand flies. Vet Parasitol. 2010 May 28;170(1-2):127-30.

[262] Ferrolho J, Maia C, Gomes J, et al. Rotation of the external genitalia in male Phlebotomine sand flies (Diptera, Psychodidae) in laboratory conditions and in captured specimens in Algarve, Portugal. Acta Trop. 2015 Oct;150:1-3.

[263] Foglia Manzillo V, Gizzarelli M, Vitale F, et al. Serological and entomological survey of canine leishmaniasis in Lampedusa island, Italy. BMC Vet Res. 2018 Sep 19;14(1):286.

[264] Fotakis EA, Giantsis IA, Castells Sierra J, et al. Population dynamics, pathogen detection and insecticide resistance of mosquito and sand fly in refugee camps, Greece. Infect Dis Poverty. 2020 Mar 18;9(1):30.

[265] Frahtia-Benotmane K, Mihoubi I, Picot S. [Molecular diagnosis by real-time PCR of Leishmania isolated from the Phlebotomus vector]. Med Sante Trop. 2013 May 1;23(2):230.

[266] Fraihi W, Fares W, Perrin P, et al. An integrated overview of the midgut bacterial flora composition of Phlebotomus perniciosus, a vector of zoonotic visceral leishmaniasis in the

Western Mediterranean Basin. PLoS Negl Trop Dis. 2017 Mar;11(3):e0005484.

[267] Gaglio G, Brianti E, Napoli E, et al. Effect of night time-intervals, height of traps and lunar phases on sand fly collection in a highly endemic area for canine leishmaniasis. Acta Trop. 2014 May;133:73-7.

[268] Gaglio G, Napoli E, Arfuso F, et al. Do Different LED Colours Influence Sand Fly Collection by Light Trap in the Mediterranean? Biomed Res Int. 2018;2018:6432637.

[269] Gaglio G, Napoli E, Falsone L, et al. Field evaluation of a new light trap for phlebotomine sand flies. Acta Trop. 2017 Oct;174:114-117.

[270] Galán-Puchades MT, Solano J, González G, et al. Molecular detection of Leishmania infantum in rats and sand flies in the urban sewers of Barcelona, Spain. Parasit Vectors. 2022 Jun 16;15(1):211.

[271] Gálvez R, Descalzo MA, Miró G, et al. Seasonal trends and spatial relations between environmental/meteorological factors and leishmaniosis sand fly vector abundances in Central Spain. Acta Trop. 2010 Jul-Aug;115(1-2):95-102.

[272] Gálvez R, Montoya A, Cruz I, et al. Latest trends in Leishmania infantum infection in dogs in Spain, Part I: mapped seroprevalence and sand fly distributions. Parasit Vectors. 2020 Apr 21;13(1):204.

[273] Gherbi R, Bounechada M, Latrofa MS, et al. Phlebotomine sand flies and Leishmania species in a focus of cutaneous leishmaniasis in Algeria. PLoS Negl Trop Dis. 2020 Feb;14(2):e0008024.

[274] Ghrab J, Rhim A, Bach-Hamba D, et al. Phlebotominae (Diptera: Psychodidae) of human leishmaniosis sites in Tunisia. Parasite. 2006 Mar;13(1):23-33.

[275] Gijón-Robles P, Abattouy N, Merino-Espinosa G, et al. Understanding the factors that determine the emergence of anthroponotic cutaneous leishmaniasis due to Leishmania tropica in Morocco: Density and mitochondrial lineage of Phlebotomus sergenti in endemic and free areas of leishmaniasis. Transbound Emerg Dis. 2022 Jul;69(4):1912-1921.

[276] Gijón-Robles P, Gómez-Mateos M, Corpas-López E, et al. Morphology does not allow differentiating the species of the Phlebotomus perniciosus complex: Molecular characterization and investigation of their natural infection by Leishmania infantum in Morocco. Zoonoses Public Health. 2023 Sep;70(6):555-567.

[277] Gómez-Saladín E, Doud CW, Maroli M. Short report: surveillance of Leishmania sp. among

sand flies in Sicily (Italy) using a fluorogenic real-time polymerase chain reaction. Am J Trop Med Hyg. 2005 Feb;72(2):138-41.

[278] González E, Jiménez M, Hernández S, et al. Phlebotomine sand fly survey in the focus of leishmaniasis in Madrid, Spain (2012-2014): seasonal dynamics, Leishmania infantum infection rates and blood meal preferences. Parasit Vectors. 2017 Aug 1;10(1):368.

[279] González E, Molina R, Iriso A, et al. Opportunistic feeding behaviour and Leishmania infantum detection in Phlebotomus perniciosus females collected in the human leishmaniasis focus of Madrid, Spain (2012-2018). PLoS Negl Trop Dis. 2021 Mar;15(3):e0009240.

[280] Gradoni L, Ferroglio E, Zanet S, et al. Monitoring and detection of new endemic foci of canine leishmaniosis in northern continental Italy: An update from a study involving five regions (2018-2019). Vet Parasitol Reg Stud Reports. 2022 Jan;27:100676.

[281] Guernaoui S, Boumezzough A, Laamrani A. Altitudinal structuring of sand flies (Diptera: Psychodidae) in the High-Atlas mountains (Morocco) and its relation to the risk of leishmaniasis transmission. Acta Trop. 2006 Mar;97(3):346-51.

[282] Guernaoui S, Boussaa S, Pesson B, et al. Nocturnal activity of phlebotomine sandflies (Diptera: Psychodidae) in a cutaneous leishmaniasis focus in Chichaoua, Morocco. Parasitol Res. 2006 Feb;98(3):184-8.

[283] Guernaoui S, Pesson B, Boumezzough A, et al. Distribution of phlebotomine sandflies, of the subgenus Larroussius, in Morocco. Med Vet Entomol. 2005 Mar;19(1):111-5.

[284] Halada P, Hlavackova K, Risueño J, et al. Effect of trapping method on species identification of phlebotomine sandflies by MALDI-TOF MS protein profiling. Med Vet Entomol. 2018 Sep;32(3):388-392.

[285] Huguenin A, Pesson B, Kaltenbach ML, et al. MALDI-TOF MS Limits for the Identification of Mediterranean Sandflies of the Subgenus Larroussius, with a Special Focus on the Phlebotomus perniciosus Complex. Microorganisms. 2022 Oct 28;10(11).

[286] Iatta R, Zatelli A, Laricchiuta P, et al. Leishmania infantum in Tigers and Sand Flies from a Leishmaniasis-Endemic Area, Southern Italy. Emerg Infect Dis. 2020 Jun;26(6):1311-1314.

[287] Jaouadi K, Bettaieb J, Bennour A, et al. Blood Meal Analysis of Phlebotomine Sandflies (Diptera: Psychodidae: Phlebotominae) for Leishmania spp. Identification and Vertebrate Blood Origin, Central Tunisia, 2015-2016. Am J Trop Med Hyg. 2018 Jan;98(1):146-149.

[288] Jaouadi K, Bettaieb J, Bennour A, et al. First Report on Natural Infection of Phlebotomus sergenti with Leishmania tropica in a Classical Focus of Leishmania major in Tunisia. Am J Trop Med Hyg. 2017 Jul;97(1):291-294.

[289] Jiménez M, González E, Iriso A, et al. Detection of Leishmania infantum and identification of blood meals in Phlebotomus perniciosus from a focus of human leishmaniasis in Madrid, Spain. Parasitol Res. 2013 Jul;112(7):2453-9.

[290] Kaabi B, Zhioua E. Modeling and comparative study of the spread of zoonotic visceral leishmaniasis from Northern to Central Tunisia. Acta Trop. 2018 Feb;178:19-26.

[291] Kabbout N, Merzoug D, Chenchouni H. Ecological Status of Phlebotomine Sandflies (Diptera: Psychodidae) in Rural Communities of Northeastern Algeria. J Arthropod Borne Dis. 2016 Mar;10(1):24-38.

[292] Karimian F, Koosha M, Choubdar N, et al. Comparative analysis of the gut microbiota of sand fly vectors of zoonotic visceral leishmaniasis (ZVL) in Iran; host-environment interplay shapes diversity. PLoS Negl Trop Dis. 2022 Jul;16(7):e0010609.

[293] Kavur H, Arikan H, Ozbel Y. Phlebotomus halepensis (Diptera: Psychodidae) Vectorial Capacity in Afyon and Nigde Province, Turkey. J Med Entomol. 2018 Feb 28;55(2):317-322.

[294] Kavur H, Artun O. Geographical Information Systems in Determination of Cutaneous Leishmaniasis Spatial Risk Level Based on Distribution of Vector Species in Imamoglu Province, Adana. J Med Entomol. 2017 Sep 1;54(5):1175-1182.

[295] Kavur H, Eroglu F, Evyapan G, et al. Entomological Survey for Sand Fly Fauna in Imamoglu Province (Cutaneous Leishmaniasis Endemic Region) of Adana, Turkey. J Med Entomol. 2015 Sep;52(5):813-8.

[296] Kravchenko V, Wasserberg G, Warburg A. Bionomics of phlebotomine sandflies in the Galilee focus of cutaneous leishmaniasis in northern Israel. Med Vet Entomol. 2004 Dec;18(4):418-28.

[297] Latrofa MS, Dantas-Torres F, Weigl S, et al. Multilocus molecular and phylogenetic analysis of phlebotomine sand flies (Diptera: Psychodidae) from southern Italy. Acta Trop. 2011 Aug;119(2-3):91-8.

[298] Latrofa MS, Iatta R, Dantas-Torres F, et al. Detection of Leishmania infantum DNA in phlebotomine sand flies from an area where canine leishmaniosis is endemic in southern Italy. Vet Parasitol. 2018 Apr 15;253:39-42.

[299] Layouni S, Remadi L, Chaâbane-Banaoues R, et al. Identification of cuticle and midgut fungal microflora of phlebotomine sandflies collected in Tunisia. Arch Microbiol. 2023 Jan 12;205(2):64.

[300] Lisi O, D'Urso V, Vaccalluzzo V, et al. Persistence of phlebotomine Leishmania vectors in urban sites of Catania (Sicily, Italy). Parasit Vectors. 2014 Dec 9;7:560.

[301] Llanes-Acevedo IP, Arcones C, Gálvez R, et al. DNA sequence analysis suggests that cytb-nd1 PCR-RFLP may not be applicable to sandfly species identification throughout the Mediterranean region. Parasitol Res. 2016 Mar;115(3):1287-95.

[302] Maia C, Afonso MO, Neto L, et al. Molecular detection of Leishmania infantum in naturally infected Phlebotomus perniciosus from Algarve region, Portugal. J Vector Borne Dis. 2009 Dec;46(4):268-72.

[303] Maia C, Dionísio L, Afonso MO, et al. Leishmania infection and host-blood feeding preferences of phlebotomine sandflies and canine leishmaniasis in an endemic European area, the Algarve Region in Portugal. Mem Inst Oswaldo Cruz. 2013 Jun;108(4):481-7.

[304] Maia C, Parreira R, Cristóvão JM, et al. Exploring the utility of phylogenetic analysis of cytochrome oxidase gene subunit I as a complementary tool to classical taxonomical identification of phlebotomine sand fly species (Diptera, Psychodidae) from southern Europe. Acta Trop. 2015 Apr;144:1-8.

[305] Manseur H, Hachid A, Khardine AF, et al. First Isolation of Punique Virus from Sand Flies Collected in Northern Algeria. Viruses. 2022 Aug 17;14(8).

[306] Maresca C, Scoccia E, Barizzone F, et al. A survey on canine leishmaniasis and phlebotomine sand flies in central Italy. Res Vet Sci. 2009 Aug;87(1):36-8.

[307] Maroli M, Rossi L, Baldelli R, et al. The northward spread of leishmaniasis in Italy: evidence from retrospective and ongoing studies on the canine reservoir and phlebotomine vectors. Trop Med Int Health. 2008 Feb;13(2):256-64.

[308] Martin E, Varotto Boccazzi I, De Marco L, et al. The mycobiota of the sand fly Phlebotomus perniciosus: Involvement of yeast symbionts in uric acid metabolism. Environ Microbiol. 2018 Mar;20(3):1064-1077.

[309] Martín-Sánchez J, Torres-Medina N, Corpas-López V, et al. Vertical transmission may play a greater role in the spread of Leishmania infantum in synanthropic Mus musculus rodents than

previously believed. Transbound Emerg Dis. 2020 May;67(3):1113-1118.

[310] Matsumoto K, Izri A, Dumon H, et al. First detection of Wolbachia spp., including a new genotype, in sand flies collected in Marseille, France. J Med Entomol. 2008 May;45(3):466-9.

[311] Melaun C, Krüger A, Werblow A, et al. New record of the suspected leishmaniasis vector Phlebotomus (Transphlebotomus) mascittii Grassi, 1908 (Diptera: Psychodidae: Phlebotominae)--the northernmost phlebotomine sandfly occurrence in the Palearctic region. Parasitol Res. 2014 Jun;113(6):2295-301.

[312] Mendoza-Roldan JA, Latrofa MS, Iatta R, et al. Detection of Leishmania tarentolae in lizards, sand flies and dogs in southern Italy, where Leishmania infantum is endemic: hindrances and opportunities. Parasit Vectors. 2021 Sep 8;14(1):461.

[313] Mendoza-Roldan JA, Zatelli A, Latrofa MS, et al. Leishmania (Sauroleishmania) tarentolae isolation and sympatric occurrence with Leishmania (Leishmania) infantum in geckoes, dogs and sand flies. PLoS Negl Trop Dis. 2022 Aug;16(8):e0010650.

[314] Messahel NE, Benallal KE, Halada P, et al. Identification of blood source preferences and Leishmania infection in sand flies (Diptera: Psychodidae) in north-eastern Algeria. Vet Parasitol Reg Stud Reports. 2022 Jun;31:100729.

[315] Mhaidi I, Ait Kbaich M, El Kacem S, et al. Entomological study in an anthroponotic cutaneous leishmaniasis focus in Morocco: Fauna survey, Leishmania infection screening, molecular characterization and MALDI-TOF MS protein profiling of relevant Phlebotomus species. Transbound Emerg Dis. 2022 May;69(3):1073-1083.

[316] Mhaidi I, El Kacem S, Ait Kbaich M, et al. Molecular identification of Leishmania infection in the most relevant sand fly species and in patient skin samples from a cutaneous leishmaniasis focus, in Morocco. PLoS Negl Trop Dis. 2018 Mar;12(3):e0006315.

[317] Miró G, Checa R, Montoya A, et al. Current situation of Leishmania infantum infection in shelter dogs in northern Spain. Parasit Vectors. 2012 Mar 27;5:60.

[318] Molina R, Jiménez M. Assessing the susceptibility to permethrin and deltamethrin of two laboratory strains of Phlebotomus perniciosus from Madrid region, Spain. Acta Trop. 2022 Jul;231:106453.

[319] Mollicone E, Battelli G, Gramiccia M, et al. A stable focus of canine leishmaniosis in the Bologna Province, Italy. Parassitologia. 2003 Jun;45(2):85-8.

[320] Montoya A, de Quadros LP, Mateo M, et al. LEISHMANIA INFANTUM INFECTION IN BENNETT'S WALLABIES (MACROPUS RUFOGRISEUS RUFOGRISEUS) IN A SPANISH WILDLIFE PARK. J Zoo Wildl Med. 2016 Jun;47(2):586-93.

[321] Moradi-Asl E, Saghafipour A, Rassi Y, et al. Spatial distribution and infection rate of leishmaniasis vectors (Diptera: Psychodidae) in Ardabil Province, Northwest of Iran. Asian Pac J Trop Biomed. 2019 05/28;9:181-187.

[322] Morillas-Márquez F, Díaz-Sáez V, Morillas-Mancilla MJ, et al. Phlebotomine sandflies (Diptera, Phlebotomidae) of Lanzarote Island (Canary Islands, Spain): Ecological survey and evaluation of the risk of Leishmania transmission. Acta Trop. 2017 Apr;168:16-20.

[323] Morosetti G, Bongiorno G, Beran B, et al. Risk assessment for canine leishmaniasis spreading in the north of Italy. Geospat Health. 2009 Nov;4(1):115-27.

[324] Morosetti G, Toson M, Trevisiol K, et al. Canine leishmaniosis in the Italian northeastern Alps: A survey to assess serological prevalence in dogs and distribution of phlebotomine sand flies in the Autonomous Province of Bolzano - South Tyrol, Italy. Vet Parasitol Reg Stud Reports. 2020 Jul;21:100432.

[325] Muñoz C, Martínez-de la Puente J, Figuerola J, et al. Molecular xenomonitoring and host identification of Leishmania sand fly vectors in a Mediterranean periurban wildlife park. Transbound Emerg Dis. 2019 Nov;66(6):2546-2561.

[326] Muñoz C, Pérez-Cutillas P, Berriatua E, et al. On how trap positioning affects phlebotomine sand fly density estimations. Med Vet Entomol. 2021 Sep;35(3):490-494.

[327] Nasereddin A, Ereqat S, Al-Jawabreh A, et al. Concurrent molecular characterization of sand flies and Leishmania parasites by amplicon-based next-generation sequencing. Parasit Vectors. 2022 Jul 22;15(1):262.

[328] Norouzi B, Hanafi-Bojd AA, Moin-Vaziri V, et al. An Inventory of the Sand Flies (Diptera: Psychodidae) of Rudbar County, a New Focus of Leishmaniasis in Northern Iran, with a Taxonomic Note on the Subgenus Larroussius. J Arthropod Borne Dis. 2020 Sep;14(3):302-316.

[329] Norouzi B, Hanafi-Bojd AA, Moin-Vaziri V, et al. Ecology of sand flies (Diptera: Psychodidae, Phlebotominae) in a new focus of leishmaniasis in northern Iran. Acta Trop. 2020 Dec;212:105649.

[330] Oerther S, Jöst H, Heitmann A, et al. Phlebotomine sand flies in Southwest Germany: an update

with records in new locations. Parasit Vectors. 2020 Apr 21;13(1):173.

[331] Omondi ZN, Demir S, Arserim SK. Entomological Survey of the Sand Fly Fauna of Kayseri Province: Focus on Visceral and Cutaneous Leishmaniasis in Central Anatolia, Turkey. Turkiye Parazitol Derg. 2020 Sep 4;44(3):158-163.

[332] Ortuño M, Muñoz-Hernández C, Risueño J, et al. Effect of high-volume insecticide spraying on sand fly vectors in household gardens in Spain. Zoonoses Public Health. 2023 Sep;70(6):511-522.

[333] Oshaghi MA, Ravasan NM, Hide M, et al. Phlebotomus perfiliewi transcaucasicus is circulating both Leishmania donovani and L. infantum in northwest Iran. Exp Parasitol. 2009 Nov;123(3):218-25.

[334] Otranto D, Paradies P, Lia RP, et al. Efficacy of a combination of 10% imidacloprid/50% permethrin for the prevention of leishmaniasis in kennelled dogs in an endemic area. Vet Parasitol. 2007 Mar 31;144(3-4):270-8.

[335] Özbel Y, Karakuş M, Arserim SK, et al. Molecular detection and identification of Leishmania spp. in naturally infected Phlebotomus tobbi and Sergentomyia dentata in a focus of human and canine leishmaniasis in western Turkey. Acta Trop. 2016 Mar;155:89-94.

[336] Pandey K, Pant S, Kanbara H, et al. Molecular detection of Leishmania parasites from whole bodies of sandflies collected in Nepal. Parasitol Res. 2008 Jul;103(2):293-7.

[337] Parvizi P, Fardid F, Soleimani S. Detection of a New Strain of Wolbachia pipientis in Phlebotomus perfiliewi transcaucasicus, a Potential Vector of Visceral Leishmaniasis in North West of Iran, by Targeting the Major Surface Protein Gene. J Arthropod Borne Dis. 2013;7(1):46-55.

[338] Pombi M, Giacomi A, Barlozzari G, et al. Molecular detection of Leishmania (Sauroleishmania) tarentolae in human blood and Leishmania (Leishmania) infantum in Sergentomyia minuta: unexpected host-parasite contacts. Med Vet Entomol. 2020 Dec;34(4):470-475.

[339] Prudhomme J, Mazza T, Hagen S, et al. New Microsatellite Markers for Genetic Studies on Sergentomyia schwetzi (Diptera: Psychodidae): A Suspected Vector of Leishmania infantum (Trypanosomatida: Trypanosomatidae) in the Canine Leishmaniasis Focus of Mont-Rolland, Senegal. J Med Entomol. 2022 Nov 16;59(6):2170-2175.

[340] Prudhomme J, Rahola N, Toty C, et al. Ecology and spatiotemporal dynamics of sandflies in the

Mediterranean Languedoc region (Roquedur area, Gard, France). Parasit Vectors. 2015 Dec 18;8:642.

[341] Rassi Y, Moradi-Asl E, Vatandoost H, et al. Insecticide Susceptibility Status of Wild Population of Phlebotomus kandelakii and Phlebotomus perfiliewi transcaucasicus Collected from Visceral Leishmaniasis Endemic Foci in Northwestern Iran. J Arthropod Borne Dis. 2020 Sep;14(3):277-285.

[342] Rassi Y, Sanei Dehkordi A, Oshaghi MA, et al. First report on natural infection of the Phlebotomus tobbi by Leishmania infantum in northwestern Iran. Exp Parasitol. 2012 Jul;131(3):344-9.

[343] Reale S, Torina A, Sole M, et al. Fluorescence-based detection of Leishmania infantum DNA in phlebotomus vectors. Ann N Y Acad Sci. 2008 Dec;1149:334-6.

[344] Remadi L, Chargui N, Jiménez M, et al. Molecular detection and identification of Leishmania DNA and blood meal analysis in Phlebotomus (Larroussius) species. PLoS Negl Trop Dis. 2020 Mar;14(3):e0008077.

[345] Risueño J, Muñoz C, Pérez-Cutillas P, et al. Understanding Phlebotomus perniciosus abundance in south-east Spain: assessing the role of environmental and anthropic factors. Parasit Vectors. 2017 Apr 19;10(1):189.

[346] Rossi E, Bongiorno G, Ciolli E, et al. Seasonal phenology, host-blood feeding preferences and natural Leishmania infection of Phlebotomus perniciosus (Diptera, Psychodidae) in a high-endemic focus of canine leishmaniasis in Rome province, Italy. Acta Trop. 2008 Feb;105(2):158-65.

[347] Rossi E, Rinaldi L, Musella V, et al. Mapping the main Leishmania phlebotomine vector in the endemic focus of the Mt. Vesuvius in southern Italy. Geospat Health. 2007 May;1(2):191-8.

[348] Sáez VD, Morillas-Márquez F, Merino-Espinosa G, et al. Phlebotomus langeroni Nitzulescu (Diptera, Psychodidae) a new vector for Leishmania infantum in Europe. Parasitol Res. 2018 Apr;117(4):1105-1113.

[349] Sawalha SS, Ramlawi A, Sansur RM, et al. Diversity, ecology, and seasonality of sand flies (Diptera: Psychodidae) of the Jenin District (Palestinian Territories). J Vector Ecol. 2017 Jun;42(1):120-129.

[350] Signorini M, Cassini R, Drigo M, et al. Ecological niche model of Phlebotomus perniciosus, the

main vector of canine leishmaniasis in north-eastern Italy. Geospat Health. 2014 Nov;9(1):193-201.

[351] Signorini M, Drigo M, Marcer F, et al. Comparative field study to evaluate the performance of three different traps for collecting sand flies in northeastern Italy. J Vector Ecol. 2013 Dec;38(2):374-8.

[352] Şuleşco T, Erisoz Kasap O, Halada P, et al. Phlebotomine sand fly survey in the Republic of Moldova: species composition, distribution and host preferences. Parasit Vectors. 2021 Jul 21;14(1):371.

[353] Talbi FZ, El Ouali Lalami A, Fadil M, et al. Entomological Investigations, Seasonal Fluctuations and Impact of Bioclimate Factors of Phlebotomines Sand Flies (Diptera: Psychodidae) of an Emerging Focus of Cutaneous Leishmaniasis in Aichoun, Central Morocco. J Parasitol Res. 2020;2020:6495108.

[354] Talbi FZ, El Ouali Lalami A, Janati Idrissi A, et al. Leishmaniasis in central morocco: seasonal fluctuations of phlebotomine sand fly in aichoun locality, from sefrou province. Patholog Res Int. 2015;2015:438749.

[355] Talbi FZ, Taam A, El Omari H, et al. Taxonomic and Ecological Interaction of Leishmaniasis Vectors (Diptera: Psychodidae) in Sefrou Province (Middle Atlas Morocco). ScientificWorldJournal. 2022;2022:9382154.

[356] Tarallo VD, Dantas-Torres F, Lia RP, et al. Phlebotomine sand fly population dynamics in a leishmaniasis endemic peri-urban area in southern Italy. Acta Trop. 2010 Dec;116(3):227-34.

[357] Tsirigotakis N, Pavlou C, Christodoulou V, et al. Phlebotomine sand flies (Diptera: Psychodidae) in the Greek Aegean Islands: ecological approaches. Parasit Vectors. 2018 Feb 20;11(1):97.

[358] Vaselek S, Ayhan N, Oguz G, et al. Sand fly and Leishmania spp. survey in Vojvodina (Serbia): first detection of Leishmania infantum DNA in sand flies and the first record of Phlebotomus (Transphlebotomus) mascittii Grassi, 1908. Parasit Vectors. 2017 Sep 26;10(1):444.

[359] Vaselek S, Dvorak V, Hlavackova K, et al. A survey of sand flies (Diptera, Phlebotominae) along recurrent transit routes in Serbia. Acta Trop. 2019 Sep;197:105063.

[360] Vaselek S, Oguz G, Ayhan N, et al. Sandfly surveillance and investigation of Leishmania spp. DNA in sandflies in Kosovo. Med Vet Entomol. 2020 Dec;34(4):394-401.

[361] Vaziri VM, Behniafar H, Spotin A, et al. Molecular Detection of Leishmania in Wild Caught

Sand Flies of Larroussius Subgenus in Iran: Combined Use of Internal and External Morphological Characters as a Mean to Differentiate Morphologically Similar Females. Acta Parasitol. 2023 Sep;68(3):520-526.

[362] Velo E, Bongiorno G, Kadriaj P, et al. The current status of phlebotomine sand flies in Albania and incrimination of Phlebotomus neglectus (Diptera, Psychodidae) as the main vector of Leishmania infantum. PLoS One. 2017;12(6):e0179118.

[363] Velo E, Paparisto A, Bongiorno G, et al. Entomological and parasitological study on phlebotomine sandflies in central and northern Albania. Parasite. 2005 Mar;12(1):45-9.

[364] Weslati M, Ghrab J, Benabid M, et al. Diversity, Abundance and Leishmania infantum Infection Rate of Phlebotomine Sandflies in an Area with Low Incidence of Visceral Leishmaniasis in Northern Tunisia. Microorganisms. 2022 May 11;10(5).

[365] Xanthopoulou K, Anagnostou V, Ivovic V, et al. Distribution of sandflies (Diptera, Psychodidae) in two Ionian Islands and northern Greece. Vector Borne Zoonotic Dis. 2011 Dec;11(12):1591-4.

[366] Zarrouk A, Boussaa S, Belqat B. Distribution and Ecology of Phlebotomine Sand Flies (Diptera: Psychodidae) in Endemic and Nonendemic Area of Leishmaniasis in Northern Morocco. J Med Entomol. 2022 Nov 16;59(6):2120-2129.

[367] Zarrouk A, Kahime K, Boussaa S, et al. Ecological and epidemiological status of species of the Phlebotomus perniciosus complex (Diptera: Psychodidae, Phlebotominae) in Morocco. Parasitol Res. 2016 Mar;115(3):1045-51.

[368] Zhioua E, Kaabi B, Chelbi I. Entomological investigations following the spread of visceral leishmaniasis in Tunisia. J Vector Ecol. 2007 Dec;32(2):371-4.

[369] Zhioua E, Moureau G, Chelbi I, et al. Punique virus, a novel phlebovirus, related to sandfly fever Naples virus, isolated from sandflies collected in Tunisia. J Gen Virol. 2010 May;91(Pt 5):1275-83.

[370] Zivicnjak T, Martinković F, Khoury C, et al. Serological and entomological studies of canine leishmaniosis in Croatia. Veterinarski Arhiv. 2011 01/01;81.

[371] Zoghlami Z, Chouihi E, Barhoumi W, et al. Interaction between canine and human visceral leishmaniases in a holoendemic focus of Central Tunisia. Acta Trop. 2014 Nov;139:32-8.

[372] Zouirech M, Rhajaoui M, Faraj C, et al. Entomological inventory of Phlebotomine sand flies

and epidemiological study of leishmaniasis in Afourer, Azilal province, High Atlas, Morocco. Med Sante Trop. 2018 Nov 1;28(4):385-389.
